# Unlocking new possibilities in ionic thermoelectric materials: a machine learning perspective

**DOI:** 10.1093/nsr/nwae411

**Published:** 2024-11-23

**Authors:** Yidan Wu, Dongxing Song, Meng An, Cheng Chi, Chunyu Zhao, Bing Yao, Weigang Ma, Xing Zhang

**Affiliations:** Key Laboratory for Thermal Science and Power Engineering of Ministry of Education, Department of Engineering Mechanics, Tsinghua University, Beijing 100084, China; Key Laboratory of Process Heat Transfer and Energy Saving of Henan Province, School of Mechanics and Safety Engineering, Zhengzhou University, Zhengzhou 450001, China; College of Mechanical and Electrical Engineering, Shaanxi University of Science and Technology, Xi’an 710021, China; Key Laboratory of Power Station Energy Transfer Conversion and System of Ministry of Education, School of Energy Power and Mechanical Engineering, North China Electric Power University, Beijing 102206, China; Key Laboratory for Thermal Science and Power Engineering of Ministry of Education, Department of Engineering Mechanics, Tsinghua University, Beijing 100084, China; Key Laboratory for Thermal Science and Power Engineering of Ministry of Education, Department of Engineering Mechanics, Tsinghua University, Beijing 100084, China; School of Materials and Chemical Engineering, Xuzhou University of Technology, Xuzhou 221018, China; Key Laboratory for Thermal Science and Power Engineering of Ministry of Education, Department of Engineering Mechanics, Tsinghua University, Beijing 100084, China; Key Laboratory for Thermal Science and Power Engineering of Ministry of Education, Department of Engineering Mechanics, Tsinghua University, Beijing 100084, China

**Keywords:** thermoelectric conversion, ionic thermoelectric materials, machine learning, interpretable analysis

## Abstract

The high thermopower of ionic thermoelectric (*i*-TE) materials holds promise for miniaturized waste-heat recovery devices and thermal sensors. However, progress is hampered by laborious trial-and-error experimentations, which lack theoretical underpinning. Herein, by introducing the simplified molecular-input line-entry system, we have addressed the challenge posed by the inconsistency of *i*-TE material types, and present a machine learning model that evaluates the Seebeck coefficient with an *R*^2^ of 0.98 on the test dataset. Using this tool, we experimentally identify a waterborne polyurethane/potassium iodide ionogel with a Seebeck coefficient of 41.39 mV/K. Furthermore, interpretable analysis reveals that the number of rotatable bonds and the octanol-water partition coefficient of ions negatively affect Seebeck coefficients, which is corroborated by molecular dynamics simulations. This machine learning-assisted framework represents a pioneering effort in the *i*-TE field, offering significant promise for accelerating the discovery and development of high-performance *i*-TE materials.

## Teaser

This work uses machine learning to predict the Seebeck coefficients of ionic thermoelectric materials, offering a faster path to discovering high-performance materials for efficient energy conversion.

## INTRODUCTION

In recent years, ionic thermoelectric (*i*-TE) materials have garnered significant interest due to their substantial Seebeck coefficients (*S*_i_, or thermopower) of ∼10 mV/K. These values are two orders of magnitude higher than those found in conventional electronic thermoelectric (*e*-TE) materials, indicating the potential to significantly decrease the number of thermoelectric pins required for thermoelectric generators. As such, *i*-TE materials could have more practical applications in compact waste-heat recovery devices [1–4] and thermal sensors [5–7]. Typically, *i*-TE materials with elevated Seebeck coefficients consist of composite structures, incorporating matrix materials (polymers, supramolecules, or nanoparticles) and ion donors (inorganic or organic salts) [8–10], because the inclusion of matrix materials augments the Eastman entropy change [11–13], thereby enhancing the Seebeck coefficient. The diversity inherent in the composite structure of *i*-TE materials mandates an evaluation of performance across various combinations of matrix materials and ion donors, thus leading to a vast chemical space with exploration. At present, the investigation into high-performance *i*-TE materials predominantly relies on empirical, trial-and-error approaches, where researchers test diverse combinations of constituents. This method, while thorough, is notably time-intensive and restricts the breadth of potential discovery.

Two key obstacles impede the extensive assessment and advancement of high-performance *i*-TE materials. First, theoretical guidance is limited due to an incomplete comprehension of the relationship between the Seebeck coefficient and the underlying physical parameters of matrix materials and ion donors. While current knowledge encompasses the correlation between Seebeck coefficients and Eastman entropy [14], the quantifiable linkage to more foundational material properties remains elusive. Second, the development of an effective computational method for rapid estimation of Seebeck coefficients and identification of optimal *i*-TE materials is hindered by the intricate interplay among ions, solvent molecules, and matrix materials [15,16]. No computational methods have been reported that can effectively characterize Seebeck coefficients for complex *i*-TE systems, with existing reported studies limited to aqueous solutions [17–19]. Evidently, in the pursuit of advanced *i*-TE materials, existing theoretical and computational frameworks are insufficient in the pursuit of advanced *i*-TE materials, while traditional trial-and-error experiments face time-consuming and scope-limiting challenges. Therefore, a pioneering strategy is imperative to address these challenges and propel the investigation of high-performance *i*-TE materials.

Fueled by the remarkable advancements of artificial intelligence, machine learning (ML) is rapidly emerging as a versatile tool in materials science, enhancing predictions, screening, and design across various applications [20,21], which brings hope for the dilemma of *i*-TE materials development. Successes of ML in material science span a spectrum, encompassing the design of *e*-TE materials [22], prediction of catalytic performance [23], identification of high-conductivity polymers [24], and evaluation of organic solar cell efficiency [25]. Despite these successes, the application of ML paradigms to the realm of *i*-TE materials remains largely untapped. The primary hurdle in harnessing ML methods for *i*-TE materials lies in defining a universal set of input feature properties. Unlike scenarios where ML has excelled with singular material types such as crystals (as in *e*-TE materials or inorganic solar cells), small molecules (like drugs), and polymers, *i*-TE materials commonly comprise matrix materials and ion donors. Matrix materials typically include polymers, nanoparticles, and supramolecules, while ion donors are often inorganic salts or organic polyelectrolytes [4,26,27]. Consequently, identifying a set of generic features capable of characterizing the properties of all these material types is paramount. For instance, glass transition temperature, a typical polymer characterization, inadequately describes matrix properties in *i*-TE materials, due to its invalidity in the presence of matrix constituents like nanoparticles or supramolecules.

**Figure 1.**
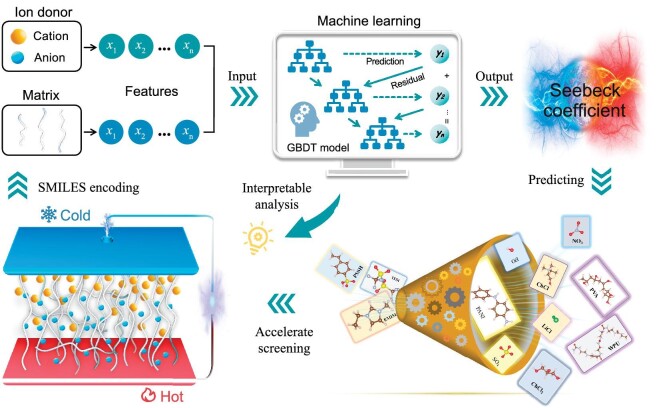
Research framework for accelerating the exploration of *i*-TE materials. *I*-TE materials experience uneven anion and cation diffusion under temperature gradients, generating an electrical potential difference to power external devices. Information on the matrix and ion donor within a specific *i*-TE material is extracted and converted into various features (*x*_1_, *x*_2_, …, *x*_n_) through SMILES encoding. An ML regression model is trained with the features to accurately evaluate the Seebeck coefficient. This model enables direct screening of *i*-TE materials. Furthermore, interpretable analysis of the ML model can provide valuable physical insights, fostering the advancement of high-performance *i*-TE materials from a more intuitive perspective.

In the present work, we propose an ML-based framework to accelerate the discovery of novel *i*-TE materials with large Seebeck coefficients (Fig. 1). To address the challenges in exploring *i*-TE materials utilizing ML techniques, we employ the simplified molecular-input line-entry system [28] (SMILES) strings as an effective encoding method for both matrix materials and ion donors. SMILES strings are found apt for representing various molecules, ionic compounds, and polymers encoded through monomers [29,30]. Subsequently, the features derived from the SMILES strings are utilized as input to train a high-precision ML regression model capable of predicting the Seebeck coefficients. Using this model, a series of recommended potential *i*-TE materials with large Seebeck coefficients are identified from a broad range of matrix and ion donor combinations. Upon experimental validation of the *i*-TE system projected to possess the largest Seebeck coefficient, in a mixture of waterborne polyurethane (WPU) and potassium iodide, we observe a positive-type Seebeck coefficient of 41.39 mV/K, nearly reaching the reported maximum. Furthermore, we utilize interpretable analysis methods to comprehend the ML model and pinpoint influential features on the Seebeck coefficient, thereby enhancing understanding of physical mechanisms within *i*-TE materials and inspiring the discovery of superior materials. The findings suggest that our ML-based framework expedites the development of *i*-TE materials via high-throughput predictions using high-accuracy ML models and enlightening information gleaned from interpretable analysis methods.

## RESULTS

### Dataset building

The Seebeck coefficient of the *i*-TE material is defined as the negative value of the voltage difference divided by the temperature difference between the cold and hot ends (${{S}_{\mathrm{i}}} = - {\mathrm{\Delta }}V/{\mathrm{\Delta }}T$). Experimental *S*_i_ values were collected from published literature, yielding a dataset of 51 samples of *i*-TE materials mostly comprising one matrix and one ion donor. The dataset encompasses 16 matrix materials and 35 ion donors, with *S*_i_ distribution displayed in Fig. S1. While constructing the dataset, we solely focused on *i*-TE composite components, as factors like electrode materials and humidity are not consistently documented. Therefore, ML-predicted values reflect ‘optimal performance,’ requiring experimental validation to identify ideal conditions.

**Figure 2.**
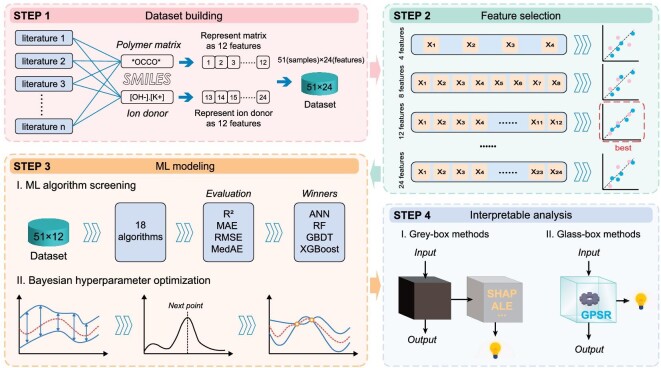
Workflow diagram for the ML-based research. It contains four major parts: dataset building (red), feature selection (green), ML modeling (orange), and interpretable analysis (cyan).

Following data collection, matrix and ion donor components must be converted to a ML-compatible format. The dataset should include matrix and ion donor properties, achieved through a feature extraction procedure. As shown in Step 1 in Fig. 2, the chemical structures of matrix materials and ion donors, representing atom arrangements and chemical bonds, are encoded as SMILES strings. The employment of SIMLES strings for encoding *i*-TE materials is found to be particularly appropriate. The main material types of matrix materials and ion donors in *i*-TE composites are molecules, polymers, inorganic salts, and organic salts. SMILES strings linearly present molecules by detailing atoms and bonds. For example, the SMILES string for phenol (C_6_H_6_O) is ‘C1=CC=C(C=C1)O’, where ‘C’ and ‘O’ represent aliphatic carbon and oxygen atoms, respectively, and ‘=’ denotes a double bond. The same digit, ‘1’ here, signifies the start and end of a ring closure, with the side chain enclosed in brackets. The asterisk (*) symbol in SMILES indicates attachment points for the repeating units of polymers (Fig. S2). Inorganic and organic salts are described by splitting the cation and anion with a period (.), like in the SMILES string of sodium acetate ‘CC(=O)[O−].[Na+]’. SMILES strings are thus key in implementing subsequent steps, resolving matrix or ion donor species inconsistencies in *i*-TE materials.

Subsequently, SMILES strings should be further converted into structured data for ML model readability. Notably, a ML method adhering to pertinent physics typically needs less training data than naive models. Therefore, SMILES strings are converted into relevant molecular descriptors (features) with a robust correlation to the Seebeck coefficients of *i*-TE materials based on fundamental physical understanding. We select 12 features that are potentially tied to *i*-TE performance from the 208 features provided by *RDKit* [31], as listed in Table 1. The reasons for choosing these 12 features and the rules for computing them are given in Note S1. The initial dataset comprised 12 matrix descriptors and 12 ion donor descriptors, yielding a 51 × 24 size (Step 1, Fig. 2), along with one target value (*S*_i_). The name of features includes a trailing label, with ‘1’ indicating matrix properties and ‘2’ for ion donor properties (e.g. ‘RB1’ and ‘RB2’ denote the number of rotatable bonds of the matrix and ion donor, respectively).

**Table 1.** The abbreviations and meanings of the twelve extracted features.

**Abbreviation**	**Name in *RDkit***	**Description**	**Unit**
MW	MolWt	The average molecular weight of the molecule	u
qed	qed	Quantitative Estimate of Drug-likeness, i.e. the weighted sum of ADS mapped properties	
VE	NumValenceElectrons	The number of valence electrons the molecule has	
BJ	BalabanJ	Balaban's J value for a molecule, a topological index meant to quantify ‘complexity’ of molecules.	
TS	TPSA	Topological polar surface area using N, O, S, P polar contributions	Å [2]
SP	FractionCSP3	Fraction of C atoms that are sp3 hybridized	
HA	NumHAcceptors	Number of acceptor atoms for H-bonds (N, O, F)	
HD	NumHDonors	Number of donor atoms for H-bonds (N and O)	
RB	NumRotatableBonds	Number of rotatable bonds	
MLP	MolLogP	Moriguchi octanol-water partition coefficient (logP)	
MR	MolMR	Wildman-Crippen molar refractivity value	
fh	fr_halogen	Number of halogens	

### ML model development and application

**Figure 3.**
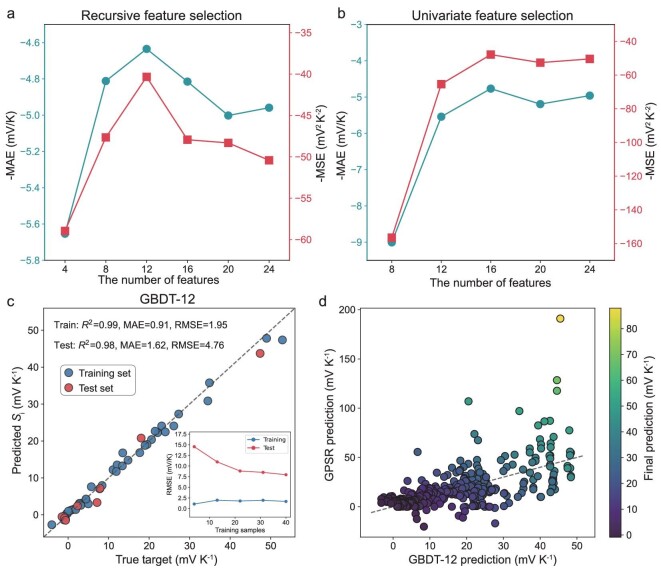
Optimization of ML models for rapid screening of *i*-TE materials. Plots of -MAE (mV/K) and -MSE (mV^2^ K^−2^) [2] of test sets with different number of input features in the GBDT-24 model using methods of (a) recursive feature elimination and (b) univariate feature selection. (c) The prediction performance of the GBDT-12 model after Bayesian hyperparameter optimization, where *R*^2^ is the coefficient of determination, and the subplot indicates the convergence of the learning curve. (d) Predictions of the Seebeck coefficient of 560 new *i*-TE materials where the color indicates the final prediction after the weighted average of GBDT-12, XGBoost-12, and GPSR. The slope of the oblique dashed line is 1.

In order to expedite *i*-TE materials development through ML, a high-precision regression model for evaluating the performance of *i*-TE materials is required. Typically, a ML model faces the ‘curse of dimensionality’ in large feature spaces due to data sparsity, overfitting, and increased computation time [32]. Consequently, feature selection is a crucial step before ML model development and optimization (Step 2, Fig. 2). To assess the performance of a ML model before feature selection, a pre-training model, gradient boosting decision trees (GBDT) [33], is trained with hyperparameters obtained by the grid search method (see Methods), as shown in Fig. S3. With 24 input features, this model is termed ‘GBDT-24’. Herein, a feature selection step based on methods of recursive feature elimination and univariate feature selection is performed (see Note S2). The effectiveness of feature selection is accessed by the negative mean absolute error (−MAE) and negative mean squared error (−MSE) in test set using 5-fold cross-validation as scoring metrics (Fig. 3a and b). The recursive feature elimination method yields maximum accuracy with 12 features, while univariate selection displays a converging performance trend with ≥12 features. Consequently, the number of input features was reduced from 24 to 12. To avert overfitting due to high correlation, Pearson correlation (Fig. S4) between variables is also calculated, aiding in the selection of the final 12 features—MW1, qed1, BJ1, HA1, MLP1, MR1, MW2, VE2, TS2, HD2, RB2, and MLP2.  Figures S5 and S6 depict their distribution and joint distribution. Employing data with 12 selected features, the pre-trained model GBDT-24 demonstrates significant improvement in generalization capability (Fig. S7).

The optimal ML regression model is trained through a systematic process. First, algorithm screening is conducted (Step 3, Fig. 2), with 4 superior algorithms chosen from an initial cohort of 18 candidates (Table S1). These selected models then undergo meticulous optimization utilizing Bayesian hyperparameter optimization techniques (Tables S2–5). A comparison of the MAE and root mean square error (RMSE) for each model within a 5-fold cross-validation framework indicates that the GBDT-12 model outperforms the others (Table S6). The ‘12’ in GBDT-12 refers to the 12 input features. In Fig. 3c, red points denote unseen test set data, and the test *R*^2^ value is determined to be 0.98. The subplot in Fig. 3c vividly portrays the gradual decrease in RMSE deviances of the test learning curve as the training size increases. This observation confirms the effectiveness of the training process of GBDT-12 in circumventing overfitting issues. Remarkably, high accuracy is attained with only 43 training samples, underscoring the benefits of integrating prior physics into ML models, by representing matrix and ion donors with SMILES strings and transforming them into 12 structural, physical, and chemical properties as input features. Adopting this strategy allows for the rapid evaluation of Seebeck coefficients for *i*-TE materials within a controlled error. Detailed information pertaining to this section can be found in the Methods section and Note S3.

One of the primary goals of this work is to employ ML techniques to forecast *i*-TE materials that possess superior Seebeck coefficients compared to those already documented. However, the extrapolation reliability of the GBDT model is often insufficient beyond the training set range. The extrapolation performance of GBDT-12 is assessed by using a test set with Seebeck coefficients above 40 mV/K and training set with the remaining samples. Results in Fig. S8 show that the predicted values for the test set are close to the maximum values in the training set but not the true values, implying the limitation of GBDT-12 in forecasting beyond the dataset. Nonetheless, GBDT-12 remains valuable for identifying novel *i*-TE materials with notable Seebeck coefficients. Caution is warranted when predicting near the maximum training set value for new matrix-ion donor combinations, as actual Seebeck coefficients may surpass predictions. In such cases, experimental validation is advised, as surprises may arise.

Symbolic regression methods, such as genetic programming symbolic regression (GPSR), enable extrapolation [34,35]. In this study, the GPSR model was employed to combine 12 input features via mathematical operators (+, −, ×, ÷, $\sqrt{} \ $ and *log*) to identify simple mathematical expressions for predicting Seebeck coefficients (Fig. S9). Disrupting combinations of matrix and ion donors in the original dataset generates 560 new combinations, whose Seebeck coefficients are predicted by GBDT-12, XGBoost-12, and GPSR models. Final predictions are weighted and averaged based on the sum of squared residuals *R*^2^ from the three models. Figure 3d showcases successful Seebeck coefficient extrapolation via GPSR, with predictions aligning well with those from GBDT-12. Despite modest prediction accuracy, GPSR proves a valuable supplementary tool in evaluating high Seebeck coefficient *i*-TE materials.

Combining the evaluation results of the above three ML models, we list the ten with the highest Seebeck coefficients among 560 matrix and ion donor combinations in Table S7. These potential high-performance *i*-TE materials warrant prioritization in future theoretical and experimental investigations.

### Experimental verification

**Figure 4.**
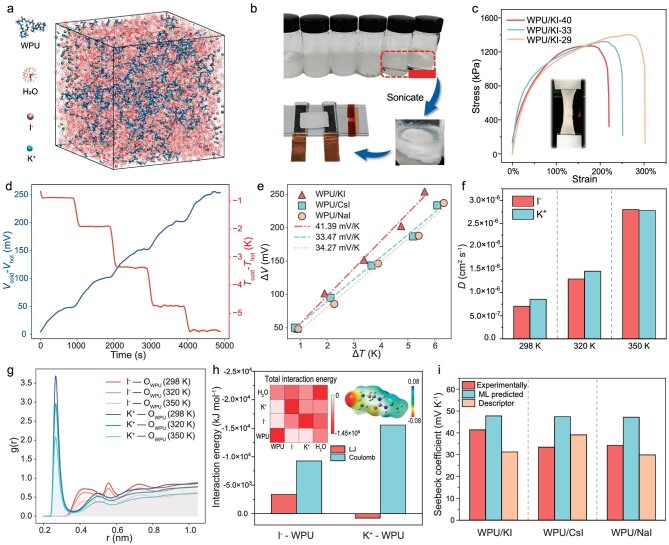
Experimental verification results and mechanism explanation. (a) The MD snapshot of WPU/KI ionogel. (b) Steps in the experimental preparation of WPU/KI ionogel, where the upper part, from left to right, shows a gradual increase in KI content (5, 10, 15, 20, 30, and 40 wt%). (c) Tensile stress-strain curves. The inset is a photo of the WPU/KI-40 during tensile testing. (d) The plot of the measured Δ*V*-Δ*T* curves. (e) The plot of Seebeck coefficient fitting curves of Δ*V*-Δ*T*. (f) The calculated diffusion coefficient of I^−^ and K^+^ in WPU/KI systems. (g) The RDFs of I^−^-O_WPU_ and K^+^-O_WPU_ at 298 K, 320 K and 350 K. (h) Plots of the interaction energies of I^−^ and K^+^ with WPU chains, where red indicates LJ weak interactions and blue indicates Coulomb electrostatic interactions. The insets, respectively, depict the interactions between different components in MD simulations and the electrostatic potential distribution of WPU in aqueous solution computed using quantum chemical methods, where red and blue colors indicate low and high electrostatic potentials, respectively. (i) Comparison of experimentally measured Seebeck coefficients with ML and descriptor predictions.

To assess the practical performance of *i*-TE systems identified via ML, we experimentally validate the top three systems listed in Table S7. These three systems share similar compositions and structures, all being WPU-based p-type ionogels composed of WPU polymers and iodide inorganic salts. This section focuses on the thermoelectric performance of WPU ionogels containing potassium iodide (WPU/KI), shown in Fig. 4a. Results of the ionogels loaded with cesium iodide (CsI) and sodium iodide (NaI) are presented in the Supplementary Information. WPU/KI ionogels are prepared by blending aqueous WPU with KI solution and dispersing through ultrasonication, detailed in Note S4. The mixture shifts from an emulsion to a quasi-solid gel with increasing KI content (Fig. 4b). The stress-strain curves of ionogels with varying KI content show three phases: linear viscoelasticity, damage accumulation, and failure (Fig. 4c). Following the linear viscoelastic stage, stress reaches a critical point, leading to microcrack formation and deviation from linear stress behavior. After achieving maximum tensile strength, the ionogel enters a failure state but does not fully rupture, allowing further strain. Figure 4c reveals that increasing KI content weakens the mechanical strength and tensile properties of the gel. Thermoelectric tests on the system containing 40 wt% KI (WPU/KI-40) were performed with an in-plane temperature gradient. As shown in Fig. 4d, the potential difference between the ends steadily increases, indicating that the ionogel has a sensitive and stable temperature response (the results of WPU/CsI and WPU/NaI are shown in Fig. S10). With a temperature difference of 0 to 5.5 K, the potential difference rises from 0 to 250 mV. Fitting the temperature and potential difference data yields a Seebeck coefficient of 41.39 mV/K (Fig. 4e).

To uncover the mechanism behind the large Seebeck coefficient of the waterborne WPU/KI ionogel, we performed all-atom molecular dynamics (MD) simulations (details in Note S7). During simulation, WPU chains, ions, and water molecules gradually formed a condensed phase (Fig. S11), with excess water surrounding the polymer-ionic aggregates (Fig. 4a), aligning with experimental phenomea (Fig. 4b). The calculated diffusion coefficients at 298 K reveal that K^+^ cations diffuse 21.4% faster than I^−^ anions (Fig. 4f), contributing to the large p-type Seebeck coefficient as per the transient Seebeck coefficient expression for binary electrolytes [1,14]:


(1) \begin{eqnarray*}{{S}_{{\mathrm{td}}}} = \frac{{{{D}_ + }{{{\hat{S}}}_ + } - {{D}_ - }{{{\hat{S}}}_ - }}}{{e({{D}_ + } + {{D}_ - })}},\end{eqnarray*}

where *D* indicates the diffusion coefficient, *e* is the electron charge, $\hat{S}$ is the Eastman entropy, and subscripts indicate the cations and anions. As temperature rises, this ion diffusion difference diminishes. The radial distribution functions (RDFs) of anions and cations with oxygen atoms in WPU (O_WPU_) at different temperatures are shown in Fig. 4g. A peak is identified at a distance of 0.27 nm between K^+^ and O_WPU_. The RDF describes the likelihood of finding a pair of atoms at a specific separation relative to a random distribution of the same density [7]. The results suggest that the 0.27 nm proximity to O_WPU_ represents a potential energy minimum for migrating K^+^ ions, drastically reducing their potential energy compared to a free state. This results in a larger height of the potential barrier between neighbor migration sites, which leads to a higher activation enthalpy for K^+^ ions. The heat of transport can be represented as [36]


(2) \begin{eqnarray*}{{Q}_ \pm } = {{k}_{\mathrm{B}}}T + \Delta {{H}_ \pm },\end{eqnarray*}

and the Seebeck coefficient is related to the heat carried by the ion through [36]


(3) \begin{eqnarray*}{{S}_ \pm } = \frac{{{{Q}_ \pm }}}{{eT}}.
\end{eqnarray*}

As evidenced by Equations (2) and (3), the significant migration barrier of K^+^ ions, results in an increased activation enthalpy and heat of transport, thereby contributing to the high p-type Seebeck coefficient of WPU/KI ionogel. Additionally, as shown in Fig. 4h, calculations of the interaction energy between ions and WPU chains indicate a robust Coulomb interaction between K^+^ ions and WPU, corroborating the results obtained from RDF analyses. Quantum chemical analysis of the electrostatic potential of WPU indicates low potentials near O_WPU_ atoms, which enhances the electrostatic attraction to K^+^ cations (inset in Fig. 4h) [37]. These results provide further insight into the substantial Seebeck coefficient observed in WPU/KI, with such pronounced ion-environment interactions typically leading to elevated Seebeck coefficients [38].

Figure 4i compares the Seebeck coefficients of the three systems predicted by ML models and the descriptor with those measured experimentally. Notably, the WPU and iodide systems represent a novel class of *i*-TE materials, with their Seebeck coefficients surpassing those of 94% of the systems in the original dataset. It should also be noted that the dataset comprises solely the composition information of matrix and ionic donors, omitting variables like electrode materials and humidity. Thus, predictions from the ML model represent optimal scenarios achievable under varying external conditions. Accordingly, to attain maximal Seebeck coefficients, experiments should explore diverse electrode materials and environmental humidity, broadening the scope of application and validity of the findings.

### Model interpretability

In this section, the intrinsic relationships between each feature and the Seebeck coefficient are revealed through interpretable ML approaches. Based on these relationships, researchers are expected to gain valuable insights, which is the second path of using ML to explore high-performance *i*-TE materials as described in Fig. 1. Two interpretable ML methods, grey-box and glass-box, are presented in this analysis.

**Figure 5.**
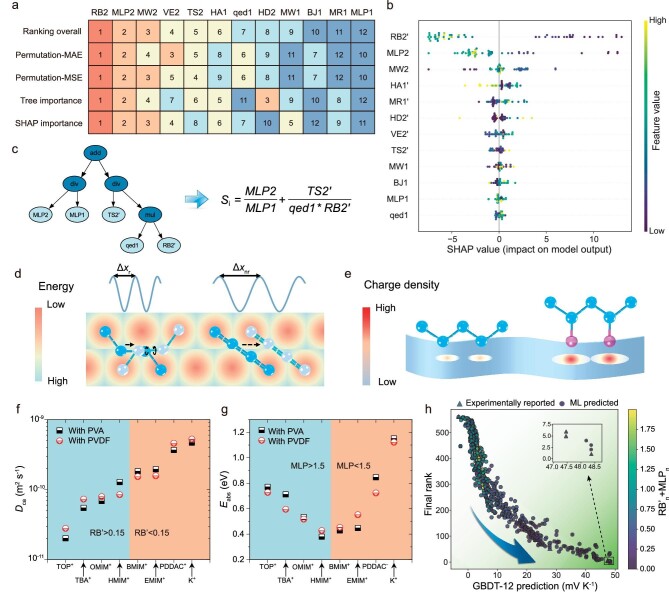
Interpretable analysis results and explanations. (a) Heatmap and ranking of the importance of each feature, where the first row shows the overall ranking and rows 2–4 correspond to the four distinct methods to calculate feature importance. (b) SHAP summary plot of 12 input features after conversion in the GBDT-12 model. The horizontal coordinate of each sample point is the SHAP value (mV/K) of each feature. Scatter points to the left of the vertical axis indicate an inverse effect on the ionic Seebeck coefficient, while those to the right indicate a positive effect. The color of the scatter represents the magnitude of feature values. (c) Plot of the tree building process of the GPSR model. (d) Comparison of the jumping distances of cations with and without rotational bonds. (e) Comparison of charge polarizations in cations without and with polar bonds. (f, g) Diffusion coefficients, *D*_ca_, and adsorption energy, *E*_abs_, of cations varying with RB and MLP. (h) ML predicted ionic Seebeck coefficients with the varying color representing the sum of RB and MLP after normalization, respectively.

Grey-box methods are post-hoc analysis techniques that extract interpretable information from black-box ML models [23]. Herein, the best-performing GBDT-12 model served as the target black-box model. Specifically, four methods were utilized to determine feature importance: permutation feature importance [39] using MAE and MSE as loss functions, tree-based feature importance [40], and Shapley additive explanations (SHAP) [41] feature importance (see Note S5, and Fig. S12). Figure 5a compares the four feature importance calculation results, with rows representing distinct methods and numbers indicating the ranking of each feature. The first row shows the overall ranking, revealing that the number of rotatable bonds and octanol-water partition coefficient of ion donors, RB2 and MLP2, greatly influence the Seebeck coefficient.

To further evaluate the quantitative and directional effect of each feature on the Seebeck coefficient, we used the SHAP technique, a grey-box method based on cooperative game theory [41]. Before calculating SHAP, the extension quantities in the 12 features were converted into intensity quantities to circumvent misleading results arising from inconsistencies in molecular or monomer size, marking the transformed features with a tail marker (e.g. RB2 to RB2'). Fig. 5b summarizes the SHAP interpretation of the GBDT-12 model. Each scatter point corresponds to an *i*-TE material in the dataset, with yellow signifying higher feature values and blue representing lower values. The negative or positive contributions of each feature can be discerned through their respective SHAP values. In the case of RB2', it emerges as the most crucial feature, where most of its higher values exert a substantial positive impact on the Seebeck coefficients, while lower values contribute negatively. MLP2 exhibits similar behavior, which is further confirmed by SHAP heatmap (Fig. S13). To further substantiate this observation, accumulated local effects (ALEs) [42] are also used to explore the quantitative effect of each feature on the *S*_i_ (Fig. S14), and the results are generally consistent with the SHAP analysis.

Although the above grey-box methods can provide valuable information, they also have a certain possibility to distort black-box model behavior [43,44]. In contrast, glass-box methods, such as GPSR, utilize symbolic regression to identify hidden data structures, enforce modularity or causal structure in predictions, and discover straightforward analytical expressions linking input variables to target properties [21]. GPSR helped identify a Seebeck coefficient descriptor (${{S}_{\mathrm{i}}} = \frac{{MLP2}}{{MLP1}} + \frac{{TS2^{\prime}}}{{qed1*RB2^{\prime}}}$) after 20 rounds of comparing 2000 genetic algorithm-generated expressions. Fig. 5c illustrates the descriptor tree building process of the GPSR model. The predicted Seebeck coefficients by the descriptor are not as accurate as the black-box model, but the trends are in agreement, as shown in Fig. S15.

Through comparing descriptor expression with grey-box model conclusions, we find that lower RB2' and MLP2 values yield higher Seebeck coefficients. These findings are explored further using theoretical analysis and molecular dynamics simulations. Notably, RB2' denotes the number of rotatable bonds per atom of the ion donor and MLP2 signifies the octanol-water partition coefficient of the ion donor. Henceforth for simplicity, the ‘2’ is omitted. Considering that most ion donors in the dataset comprise large cationic groups and monoatomic anions, cations primarily contribute to RB and MLP values. Therefore, we select four cations with high RB and MLP values, shown in Fig. S16a, and four with low values, presented in Fig. S16b, from the ion donor database.

Next, we elucidate the underlying physical processes via which RB' and MLP influencing the Seebeck coefficient. From a kinetic perspective, superior ion diffusion coefficients promote an increase in the Seebeck coefficient (Eq. 1). The diffusion coefficient can be expressed as [45]


(4) \begin{eqnarray*}{{D}_{{\mathrm{ca}}}} = c{{v}^*}{{(\Delta x)}^2}\exp ( - \Delta H/{{k}_{\mathrm{B}}}T),\end{eqnarray*}

where *c* and v* are the concentration of the diffusion-mediating defect and the attempting jump frequency, and *k*_B_ and *T* denote the Boltzmann constant and temperature. Δ*x* indicates the hop distance of mobile ions (i.e. the distance between two barriers). Figure 5d illustrates that an escalation in the number of rotational bonds triggers a reduction in Δ*x*, in turn decreasing the diffusion coefficient, which is undesirable for the thermoelectric effect. From a thermodynamic viewpoint, the Seebeck coefficient exhibits proportionality to the ion migration potential (Eqs 2 and 3). The migration potential barrier is interconnected with the bond strength between the ion and the matrix material, signifying a positive correlation with the adsorption energy between the two, thereby facilitating an increase in the Seebeck coefficient. The MLP is dependent on the polarity of ions; the higher the polarity, the more hydrophilic the ion, thus decreasing the MLP. Figure 5e demonstrates a high ion polarity inciting a polarization of the matrix charge, leading to an enhanced Coulomb attraction. Hence, a low MLP, indicative of high polarity, contributes to a heightened adsorption energy.

MD methods are employed to compute diffusion coefficients and adsorption energies of eight cations in poly(vinyl alcohol) (PVA) and polyvinylidene fluoride (PVDF) matrix materials (Fig. 5f and g). It is found that smaller values of RB and MLP lead to higher diffusion capacity and adsorption energy of the ions, respectively, resulting in larger Seebeck coefficients. The contribution of MLP to the Seebeck coefficient is not unidirectional, mirroring MLP2 behavior in Fig. 5b. The procedure for MD simulations in this section is described in Note S6.

From the application point of view, the values of RB and MLP can serve as a criterion for screening optimal ion donors when exploring high-performance *i*-TE materials in experiments. Figure 5h depicts 560 data points, each representing an *i*-TE material. The horizontal axis displays the predicted values from the GBDT-12 model, while the vertical axis presents a combined ranking of Seebeck coefficient predictions from three distinct ML models. The positioning of data points towards the lower-right corner signifies larger Seebeck coefficients. The RB and MLP values of each of the 560 *i*-TE materials were normalized and then summed to the color of the points in Fig. 5h. It can be seen that the high-performance region is characterized by a small sum of RB and MLP for both experimentally reported and ML model-predicted *i*-TE materials. By considering the positioning and color-coding of these data points, promising *i*-TE materials with large Seebeck coefficients can be identified.

## DISCUSSION

Within this work, we introduce a ML-assisted framework for the expedited screening and development of superior *i*-TE materials. The ML models can utilize almost any materials representable by a SMILES string as input, such as polymers, supramolecules, nanoparticles, and organic or inorganic salts. The robust reliability of GBDT-12 predictions, with a high *R*^2^ of 0.98 in the test set, offer a reliable foundation for swift screening of large Seebeck coefficient *i*-TE materials. The GPSR model is utilized to aid in determining samples where the predicted Seebeck coefficient nears the maximum value in the training set. Utilizing the GBDT-12 and GPSR models in tandem enables the estimation of the Seebeck coefficient for unexplored *i*-TE materials. We experimentally validate the forecasts of the ML framework for high-performance *i*-TE materials—waterborne polyurethane-iodide ionogels—achieving Seebeck coefficients up to 41.39 mV/K. The concordance between experimental validation and ML predictions is, to some extent, attributable to the use of datasets derived from experimental data documented in the literature. Interpretable analysis identifies the number of rotatable bonds and octanol-water partition coefficient of ion donors as key factors influencing the Seebeck coefficient, from kinetic and thermodynamic perspectives, respectively, confirmed via molecular dynamics simulations. Establishing a direct connection between the fundamental properties and the performance parameters of *i*-TE materials offers the potential to advance the theoretical understanding of *i*-TE materials. Notably, this study focuses solely on thermodiffusion-based *i*-TE materials driven by the Soret effect, excluding thermogalvanic cells due to their distinct Seebeck coefficient generation mechanisms. Inspired by this work, it can be anticipated that future studies may explore ML methods to develop thermogalvanic cells.

As a pioneering study, this work underscores the feasibility and efficiency of employing ML techniques to investigate *i*-TE materials, unlocking new possibilities for successors to further delve into *i*-TE research using ML methods. Future advancements in rapid experimental measurements, encompassing variables like electrode material types, environmental humidity, and ion donor mass fractions, could further enhance the accuracy and applicability of the ML models. Additionally, if a simulation method for rapid evaluation of the Seebeck coefficient of *i*-TE materials is developed, it would enable swift sample labeling, eschewing sluggish experimental measurements, and generating ample data for ML. The ML perspective is not restricted solely to explore *i*-TE materials with large Seebeck coefficients, but can also be applied to other properties such as ion mobility, thermal conductivity, and energy density, provided that an appropriate training set can be generated.

## METHODS

### Machine learning models

We employed 18 different regression algorithms containing 6 liner model algorithms (Liner Regression, Ridge, Lasso, Bayesian Ridge, Automatic Relevance Determination (ARD), Stochastic Gradient Descent (SGD)), three support sector machine algorithms (Support Vector Regressor (SVR), Nu SVR, and Linear SVR), one nearest neighbor algorithms (K Neighbors Regressor), one Gaussian process algorithm (Gaussian Process Regression), one cross decomposition algorithm (Partial Least Squares (PLS) Regression), one decision tree algorithm (Decision Tree Regressor), four ensemble learning algorithms (Random Forest (RF) Regressor, Extra Trees Regressor, Gradient Boosting Decision Trees (GBDT) Regressor, and Extreme Gradient Boosting (XGBoost)), and one neural network algorithm (Artificial Neural Network (ANN)). The *Scikit-learn* [46] library is used in python for the implementation of all these ML models.

### Evaluation metrics

The prediction accuracy of a ML model is evaluated based on the mean average error (MAE), root mean squared error (RMSE), median absolute error (MedAE), and the coefficient of determination (*R*^2^), given by the formulas:


(5) \begin{eqnarray*}MAE = \frac{{\sum\nolimits_{\mathrm{i}}^n {\left| {{{y}_i} - {{{\hat{y}}}_i}} \right|} }}{n},\end{eqnarray*}

(6) \begin{eqnarray*}\textit{RMSE} = \sqrt {\frac{{\sum\nolimits_i^n {{{{\left( {{{y}_i} - {{{\hat{y}}}_i}} \right)}}^2}} }}{n}} ,\end{eqnarray*}

(7) \begin{eqnarray*}
\textit{MedAE}(y,\,\hat{y}) = \textit{median}\left( {\left| {{{y}_1} - {{{\hat{y}}}_1}} \right|,...,\left| {{{y}_1} - {{{\hat{y}}}_1}} \right|} \right),\end{eqnarray*}

(8) \begin{eqnarray*}{{R}^2} = 1 - \frac{{\sum\nolimits_i^n {{{{\left( {{{y}_i} - {{{\hat{y}}}_i}} \right)}}^2}} }}{{\sum\nolimits_i^n {{{{\left( {{{y}_i} - \bar{y}} \right)}}^2}} }},\end{eqnarray*}

where *y_i_* and ${{\hat{y}}_i}$ represent the true value (the Seebeck coefficient documented in the literature) and the ML predicted value of the *i*-th sample, respectively. $\bar{y}$ represents the average of *y_i_*, and *n* is the number of samples in the dataset. In addition, we employed cross-validation methodology [47] along with a repeated K-fold approach [48], wherein the dataset is iteratively partitioned into training and testing subsets, to estimate prediction errors and mitigate overfitting.

### Hyperparameter optimization

In this study, two hyperparameter optimization methods, namely grid search and Bayesian optimization, were employed to enhance the prediction accuracy of the model. The grid search method optimizes the model by exhaustive combinations of hyperparameters. However, with limited computational resources, exhaustive validation of intricate hyperparameters, which can span tens of thousands of combinations, is unfeasible. Bayesian optimization uses a Gaussian process to describe correlations between search space parameters and the target variable, allowing efficient exploration of hyperparameter space by selecting the most promising parameters based on past evaluations. Accordingly, with regard to the hyperparameter space for each model, we employed grid search method to address the hyperparameter optimization task of the pre-trained model and the ANN, and applied Bayesian tuning to resolve the hyperparameter optimization process of the RF, GBDT, and XGBoost. Grid search and Bayesian optimization are implemented through the *Scikit-learn* and *hyperopt* [49] packages in python, respectively.

## Supplementary Material

nwae411_Supplemental_File

## References

[1] Han C-G, Qian X, Li Q et al. Giant thermopower of ionic gelatin near room temperature. Science 2020; 368: 1091–8. 10.1126/science.aaz5045 32354840

[2] Yu B, Duan J, Cong H et al. Thermosensitive crystallization–boosted liquid thermocells for low-grade heat harvesting. Science 2020; 370: 342–6. 10.1126/science.abd6749 32913001

[3] Wang Y, Zhang Y, Xin X et al. In situ photocatalytically enhanced thermogalvanic cells for electricity and hydrogen production. Science 2020; 381: 291–6. 10.1126/science.adg0164 37471552

[4] Li T, Zhang X, Lacey SD et al. Cellulose ionic conductors with high differential thermal voltage for low-grade heat harvesting. Nat Mater 2019; 18: 608–13. 10.1038/s41563-019-0315-6 30911121

[5] Zhao D, Fabiano S, Berggren M et al. Ionic thermoelectric gating organic transistors. Nat Commun 2017; 8: 14214. 10.1038/ncomms14214 28139738 PMC5290323

[6] Zhao W, Zheng Y, Jiang M et al. Exceptional n-type thermoelectric ionogels enabled by metal coordination and ion-selective association. Sci Adv 2023; 9: eadk2098. 10.1126/sciadv.adk2098 37878706 PMC10599631

[7] Chi C, Liu G, An M et al. Reversible bipolar thermopower of ionic thermoelectric polymer composite for cyclic energy generation. Nat Commun 2023; 14: 306. 10.1038/s41467-023-36018-w 36658195 PMC9852232

[8] Wang S, Li Y, Yu M et al. High-performance cryo-temperature ionic thermoelectric liquid cell developed through a eutectic solvent strategy. Nat Commun 2024; 15: 1172. 10.1038/s41467-024-45432-7 38332129 PMC10853189

[9] Zhang D, Zhou Y, Mao Y et al. Highly antifreezing thermogalvanic hydrogels for human heat harvesting in ultralow temperature environments. Nano Lett 2023; 23: 11272–9. 10.1021/acs.nanolett.3c03818 38038230

[10] Qian X, Ma Z, Huang Q et al. Thermodynamics of ionic thermoelectrics for low-grade heat harvesting. ACS Energy Lett 2024; 9: 679–706. 10.1021/acsenergylett.3c02448

[11] Cheng H, He X, Fan Z et al. Flexible quasi-solid state ionogels with remarkable seebeck coefficient and high thermoelectric properties. Adv Energy Mater 2019; 9: 1901085. 10.1002/aenm.201901085

[12] Zhao D, Martinelli A, Willfahrt A et al. Polymer gels with tunable ionic Seebeck coefficient for ultra-sensitive printed thermopiles. Nat Commun 2019; 10: 1093. 10.1038/s41467-019-08930-7 30842422 PMC6403253

[13] Wang H, Ail U, Gabrielsson R et al. Ionic Seebeck effect in conducting polymers. Adv Energy Mater 2015; 5: 1500044. 10.1002/aenm.201500044

[14] Song D, Chi C, An M et al. Ionic Seebeck coefficient and figure of merit in ionic thermoelectric materials. Cell Rep Phys Sci 2022; 3: 101018. 10.1016/j.xcrp.2022.101018

[15] Cheng H, Ouyang J. Soret effect of ionic liquid gels for thermoelectric conversion. J Phys Chem Lett 2022; 13: 10830–42. 10.1021/acs.jpclett.2c02645 36382894

[16] Xu Y, Li Z, Wu L et al. Solvation engineering via fluorosurfactant additive toward boosted lithium-ion thermoelectrochemical cells. Nanomicro Lett 2024; 16: 72. 38175313 10.1007/s40820-023-01292-2 PMC10766582

[17] Nickel O, Ahrens-Iwers LJ, Meißner RH et al. Water, not salt, causes most of the Seebeck effect of nonisothermal aqueous electrolytes. Phys Rev Lett 2024; 132: 186201. 10.1103/PhysRevLett.132.186201 38759182

[18] Rezende Franco L, Sehnem AL, Figueiredo Neto AM et al. Molecular dynamics approach to calculate the thermodiffusion (Soret and Seebeck) coefficients of salts in aqueous solutions. J Chem Theory Comput 2021; 17: 3539–53. 10.1021/acs.jctc.1c00116 33942620

[19] Di Lecce S, Albrecht T, Bresme F. A computational approach to calculate the heat of transport of aqueous solutions. Sci Rep 2017; 7: 44833. 10.1038/srep44833 28322266 PMC5359663

[20] Butler KT, Davies DW, Cartwright H et al. A. Machine learning for molecular and materials science. Nature 2018; 559: 547–55. 10.1038/s41586-018-0337-2 30046072

[21] Batra R, Song L, Ramprasad R. Emerging materials intelligence ecosystems propelled by machine learning. Nat Rev Mater 2021; 6: 655–78. 10.1038/s41578-020-00255-y

[22] Bassman Oftelie L, Rajak P, Kalia RK et al. Active learning for accelerated design of layered materials. NPJ Comput Mater 2018; 4: 74. 10.1038/s41524-018-0129-0

[23] Esterhuizen JA, Goldsmith BR, Linic S. Interpretable machine learning for knowledge generation in heterogeneous catalysis. Nat Catal 2022; 5: 175–84. 10.1038/s41929-022-00744-z

[24] Wu S, Kondo Y, Kakimoto MA et al. Machine-learning-assisted discovery of polymers with high thermal conductivity using a molecular design algorithm. NPJ Comput Mater 2019; 5: 66. 10.1038/s41524-019-0203-2

[25] Sahu H, Rao W, Troisi A et al. Toward predicting efficiency of organic solar cells via machine learning and improved descriptors. Adv Energy Mater 2018; 8: 1801032. 10.1002/aenm.201801032

[26] Ding Y, Zhang J, Chang L et al. Preparation of high-performance ionogels with excellent transparency, good mechanical strength, and high conductivity. Adv Mater 2017; 29: 1704253. 10.1002/adma.201704253 29083496

[27] Néouze M-A, Le Bideau J, Gaveau P et al. Ionogels, new materials arising from the confinement of ionic liquids within silica-derived networks. Chem Mat 2006; 18: 3931–6. 10.1021/cm060656c

[28] Weininger D. SMILES, a chemical language and information system. 1. Introduction to methodology and encoding rules. J Chem Inf Comput Sci 1988; 28: 31–6. 10.1021/ci00057a005

[29] Cao Y, Yang ZQ, Zhang XL et al. Identifying the kind behind SMILES—Anatomical therapeutic chemical classification using structure-only representations. Brief Bioinform 2022; 23: bbac346. 10.1093/bib/bbac346 36027578

[30] Ma R, Zhang H, Luo T. Exploring high thermal conductivity amorphous polymers using reinforcement learning. ACS Appl Mater Interfaces 2022; 14: 15587–98. 10.1021/acsami.1c23610 35344333

[31] Landrum G. Rdkit documentation. https://buildmedia.readthedocs.org/media/pdf/rdkit/latest/rdkit.pdf (7 November 2024, date last accessed).

[32] Li J, Cheng K, Wang S et al. Feature selection: a data perspective. ACM Comput Surv 2017; 50: 1–45.

[33] Friedman JH. Greedy function approximation: a gradient boosting machine. Ann Stat 2001; 29: 1189–232. 10.1214/aos/1013203451

[34] Wang Y, Wagner N, Rondinelli JM. Symbolic regression in materials science. MRS Commun 2019; 9: 793–805. 10.1557/mrc.2019.85

[35] Ouyang R, Curtarolo S, Ahmetcik E et al. SISSO: a compressed-sensing method for identifying the best low-dimensional descriptor in an immensity of offered candidates. Phys Revw Mater 2018; 2: 083802. 10.1103/PhysRevMaterials.2.083802

[36] Würger A. Thermoelectric ratchet effect for charge carriers with hopping dynamics. Physl Rev Lett 2021; 126: 068001. 10.1103/PhysRevLett.126.068001 33635717

[37] Zhao W, Zheng Y, Huang A et al. Metal-halogen interactions inducing phase separation for self-healing and tough ionogels with tunable thermoelectric performance. Adv Mater 2024; 36: 2402386. 10.1002/adma.202402386 38708954

[38] Yu M, Li H, Li Y et al. Ionic thermoelectric gels and devices: progress, opportunities, and challenges. EnergyChem 2024; 6: 100123. 10.1016/j.enchem.2024.100123

[39] Fisher A, Rudin C, Dominici F. All models are wrong, but many are useful: learning a variable's importance by studying an entire class of prediction models simultaneously. J Mach Learn Res 2019; 20: 1–81. PMC8323609 34335110

[40] Hastie T, Tibshirani R, Friedman JH et al. The Elements of Statistical Learning: Data Mining, Inference, and Prediction. New York: Springer, 2017.

[41] Lundberg SM, Erion G, Chen H et al. From local explanations to global understanding with explainable AI for trees. Nat Mach Intell 2020; 2: 56–67. 10.1038/s42256-019-0138-9 32607472 PMC7326367

[42] Apley DW, Zhu J. Visualizing the effects of predictor variables in black box supervised learning models. J R Stat Soc B 2020; 82: 1059–86. 10.1111/rssb.12377

[43] Rudin C. Stop explaining black box machine learning models for high stakes decisions and use interpretable models instead. Nat Mach Intell 2019; 1: 206–15. 10.1038/s42256-019-0048-x 35603010 PMC9122117

[44] Murdoch WJ, Singh C, Kumbier K et al. Definitions, methods, and applications in interpretable machine learning. Proc Natl Acad Sci USA 2019; 116: 22071–80. 10.1073/pnas.1900654116 31619572 PMC6825274

[45] Van der Ven A, Ceder G, Asta M et al. First-principles theory of ionic diffusion with nondilute carriers. Phys Rev B 2001; 64: 184307. 10.1103/PhysRevB.64.184307

[46] Pedregosa F, Varoquaux G, Gramfort A et al. Scikit-learn: machine learning in Python. J Mach Learn Res 2011; 12: 2825–30.

[47] Hawkins DM, Basak SC, Mills D. Assessing model fit by cross-validation. J Chem Inf Comput Sci 2003; 43: 579–86. 10.1021/ci025626i 12653524

[48] Fushiki T. Estimation of prediction error by using K-fold cross-validation. Stat Comput 2021; 21: 137–46. 10.1007/s11222-009-9153-8

[49] Bergstra J, Komer B, Eliasmith C et al. Hyperopt: a Python library for model selection and hyperparameter optimization. Comput Sci Discov 2015; 8: 014008. 10.1088/1749-4699/8/1/014008

