# Supplementary material for: Unlocking new possibilities in ionic thermoelectric materials: a machine learning perspective
Source: Natl Sci Rev. 2024 Nov 23;12(1):nwae411. doi: 10.1093/nsr/nwae411 (PMC11702661; doi:10.1093/nsr/nwae411)
Supplement: nwae411_Supplemental_File [file nwae411_supplemental_file.zip › Supplementary data.pdf]

Supplementary Information

Unlocking New Possibilities in Ionic Thermoelectric Materials: A Machine Learning Perspective

Yidan Wu[1], Dongxing Song[2], Meng An[3], Cheng Chi[4], Chunyu Zhao[1], Bing Yao[1,5], Weigang Ma[1]*, and Xing Zhang[1]

[1]Key Laboratory for Thermal Science and Power Engineering of Ministry of Education, Department of Engineering Mechanics, Tsinghua University, Beijing 100084, China.

[2]Key Laboratory of Process Heat Transfer and Energy Saving of Henan Province, School of Mechanics and Safety Engineering, Zhengzhou University, Zhengzhou, Henan 450001, China.

[3]College of Mechanical and Electrical Engineering, Shaanxi University of Science and Technology, Xi'an 710021, China.

[4]Key Laboratory of Power Station Energy Transfer Conversion and System of Ministry of Education, School of Energy Power and Mechanical Engineering, North China Electric Power University, Beijing 102206, China.

[5]School of Materials and Chemical Engineering, Xuzhou University of Technology, Xuzhou, Jiangsu 221018, China.

*Corresponding author: W. G. Ma, email: maweigang@tsinghua.edu.cn

Inventory of Supplementary Information:

Supplementary Note 1. Details about input features

Supplementary Note 2. Feature selection

Supplementary Note 3. Algorithm screening

Supplementary Note 4. Experimental section

Supplementary Note 5. Model interpretation

Supplementary Note 6. Calculation of diffusion coefficients and adsorption energies

Supplementary Note 7. Computational methods

Supplementary Fig. S1. S_i kernel distribution of totally 51 *i*-TE materials.

Supplementary Fig. S2. An example of the representation of polymer matrix by SMILES encoding.

Supplementary Fig. S3. The prediction results of the pre-trained model GBDT-24.

Supplementary Fig. S4. Pearson correlation of 24 input features and S_i.

Supplementary Fig. S5. Scatterplot that summarizes the joint distribution of the six selected properties of matrix materials.

Supplementary Fig. S6. Scatterplot that summarizes the joint distribution of the six selected properties of ion donors.

Supplementary Fig. S7. The GBDT-24 model retrained with 12 selected features.

Supplementary Fig. S8. The prediction results of the GBDT-12 model using a training dataset containing samples exhibiting Seebeck coefficients below 40 mV/K.

Supplementary Fig. S9. GPSR aided forecast of Seebeck coefficients.

Supplementary Fig. S10. The plots of the measured ΔV-ΔT curves of WPU/CsI-40 and WPU/NaI-40.

Supplementary Fig. S11. Snapshots of different moments in MD simulations.

Supplementary Fig. S12. Feature importance details.

Supplementary Fig. S13. The plot of SHAP heatmap.

Supplementary Fig. S14. Accumulated local effects (ALE) chart of the twelve features.

Supplementary Fig. S15. Plots of the predicted versus true values of Seebeck coefficient by the GPSR model.

Supplementary Fig. S16. Typical cations that resulting in higher and lower ionic Seebeck coefficient respectively, where the RB and MLP values are marked.

Supplementary Fig. S17. Schematic of the steps of model construction and molecular dynamics simulations for the mixture of polymers and ionic donors.

Supplementary Fig. S18. Adsorption configurations of cations with PVA.

Supplementary Table S1. Statistical parameters in the training and test sets for 18 models.

Supplementary Table S2. The setup of hyperparameters in sklearn for ANN-12

Supplementary Table S3. The setup of hyperparameters in sklearn for RF-12

Supplementary Table S4. The setup of hyperparameters in sklearn for GBDT-12

Supplementary Table S5. The setup of hyperparameters in sklearn for XGBoost-12

Supplementary Table S6. Statistical parameters in the training and test sets for the 4 models under 10-fold cross-validation.

Supplementary Table S7. The ten matrix-ion donor combinations with the largest Seebeck coefficients predicted by GBDT-12 and XGBoost-12.

Supplementary References

Supplementary Notes

Note 1. Details about input features

This part briefly introduces the reasons for selecting the 12 original features and the calculation rules.

MW (MolWt): The value of *MolWt* equals the average molecular weight of the molecule. If the object is a polymer, the value of this feature is equal to the average molecular mass of the monomer. If the object is an inorganic salt or an organic salt, the value of this feature is equal to the sum of molecular weights of the anions and cations. Molecular weight is one of the fundamental properties of a molecule and was therefore selected.

qed: *Qed*, short for quantitative estimation of drug-likeness, was introduced by Bickerton et al. as a comprehensive descriptor that combines various molecular properties, including molecular weight, logP, topological polar surface area, hydrogen bond donors and acceptors, aromatic rings, rotatable bonds, and unwanted chemical functionalities[1]. In this study, *qed* was selected for two primary reasons. Firstly, to investigate the potential correlation between the performance of *i*-TE materials and their drug-like characteristics, especially for organic salt, which would be a significant discovery if a strong association is observed. Secondly, as a comprehensive descriptor, Qed contributes to the construction of a more comprehensive feature space for i-TE materials, enabling a more thorough analysis of their properties.

VE (NumValenceElectrons): *VE* equals the number of valence electrons the molecule has. Valence electrons play a crucial role in determining the electronic structure and bonding characteristics of a material, which in turn affect its thermal and electrical transport properties.

In some cases, an increase in the number of valence electrons can enhance the electrical conductivity of a material. This is because additional valence electrons can contribute to a higher density of charge carriers, such as electrons or holes, which are responsible for electrical conduction.

BJ (BalabanJ): *BalabanJ* is the Balaban's J value for a molecule, a topological index meant to quantify "complexity" of molecules[2]:

$$J = \frac{q}{\mu + 1} \sum_{edgesij} (S_i S_j)^{-1/2}\,, \qquad (1)$$

where, q is the number of edges in the molecular graph, S_i is the distance sums calculated as the sums over the rows or columns of the topological distance matrix of the molecule. And $\mu = q - n + 1$, where n denotes the number of atoms in the molecular graph. Higher values of Balaban's J index indicate increased molecular branching and cyclicity, which can potentially affect the charge carrier mobility and phonon scattering processes. In this paper, this feature was included into the feature set in an attempt to explore whether there is a direct relationship between the complexity of the molecule and the ionic thermoelectric properties.

TS (TPSA): *TPSA* (Topological Polar Surface Area) represents the sum of the polar surface areas of a molecule's functional groups and is an indicator of its molecular polarity and potential for interactions with other molecules[3]. In *RDKit*, the *TPSA* is calculated using the fragment-based method, which involves breaking down the molecule into fragments representing functional groups and determining their individual contributions to the polar surface area. The contributions are then summed up to obtain the total TPSA value. It was reported that matrix materials containing polar groups would give rise to a high ionic Seebeck coefficient[4,5]. Therefore, *TPSA* was included into the feature set.

SP (FractionCSP3): The *FractionCSP3* (Fraction of Carbon atoms that are SP3 hybridized) measures the ratio of carbon atoms that are SP3 hybridized (having four sigma bonds) to the total number of carbon atoms in a molecule. In *RDKit*, the *FractionCSP3* is determined by analyzing the molecular structure and identifying the carbon atoms with SP3 hybridization. The count of these carbon atoms is divided by the total count of carbon atoms in the molecule to obtain the *FractionCSP3* value. The *FractionCSP3* is an important molecular descriptor that provides information about the degree of saturation or branching in a molecule. A higher *FractionCSP3* value indicates a higher proportion of carbon atoms with tetrahedral geometry, which is typically associated with more saturated and less branched structures. Considering that the performance of *i*-TE materials may be related to the structure and packaging of the materials, the *FractionCSP3* was chosen.

HA (NumHAcceptors): *NumHAcceptors* represents the number of hydrogen bond acceptor sites in a molecule. In *RDKit*, it is calculated based on the molecular structure by identifying specific atom types that can act as hydrogen bond acceptors, such as oxygen (O) and nitrogen (N) atoms. The count of these acceptor sites provides the *NumHAcceptors* value. *NumHAcceptors* represents the count of hydrogen bond acceptor sites, indicating the ability to accept hydrogen bonds. It has been reported that the hydrogen bond between anions and cations will cause a large enthalpy change for ion transport and lead to the change of the sign of the Seebeck coefficient[6]. Consequently, *NumHAcceptors* was chosen into the feature set.

HD (NumHDonors): *NumHDonors* represents the count of hydrogen bond donor sites in a molecule. In *RDKit*, the *NumHDonors* value is determined by counting the number of eligible hydrogen bond donor sites in the molecule based on the specified atom types. Each

eligible hydrogen atom is counted as a hydrogen bond donor, contributing to the overall *NumHDonors* count. *NumHDonors* like *NumHAcceptors*, contains information about hydrogen bonding in the material and was therefore also put into the feature set.

RB (NumRotatableBonds): *NumRotatableBonds* represents the count of rotatable bonds in a molecule. It indicates the number of bonds within the molecule that can rotate freely without breaking any chemical bonds. The calculation of *NumRotatableBonds* involves analyzing the connectivity of the molecule and identifying the bonds that allow for rotational flexibility. Generally, single bonds connecting non-aromatic atoms are considered rotatable, while double or triple bonds, as well as bonds involving aromatic atoms, are considered non-rotatable. *RDKit* utilizes a heuristic algorithm to estimate the number of rotatable bonds based on the molecule's structural features. It takes into account factors such as the presence of single bonds, ring systems, and side chains to determine the potential for bond rotation. The *NumRotatableBonds* value provides information about the molecular flexibility and conformational freedom of a compound. It indicates the number of bonds that can undergo rotational motion, allowing the molecule to adopt different conformations or spatial arrangements. The presence of a higher number of rotatable bonds suggests greater flexibility and potential for structural changes. The flexibility of the ions may affect their transport properties in the surrounding and thus have an impact on the ionic thermoelectric properties, which are discussed in the main text. In view of this, *NumRotatableBonds* was put into the feature set.

MLP (MolLogP): *MolLogP* represents the logarithm of the partition coefficient, also known as the octanol-water partition coefficient (*LogP*), of a molecule[7,8]. It is a measure of

the lipophilicity or hydrophobicity of the molecule. In *RDKit*, the calculation of *MolLogP* is based on the fragment-based method, which estimates the *LogP* value by summing the contributions from individual molecular fragments[9]. The fragments are assigned specific *LogP* values based on predefined fragment contributions. The *LogP* value itself indicates the tendency of a compound to partition between an organic solvent (usually octanol) and water. It reflects the balance between the hydrophobic interactions of the molecule with the organic phase and its hydrophilic interactions with the aqueous phase. A higher *LogP* value suggests higher lipophilicity, indicating that the molecule has a greater affinity for the organic phase. Conversely, a lower *LogP* value indicates higher hydrophilicity, with a stronger preference for the aqueous phase. Water can screen the Coulomb interactions among the ions, and increase the mobilities of ions[10]. Commonly, water preferentially solvates the small anions because the bulky cations have hydrophobic moiety. The water solvation increases the size of the anions, and the anions solvated with polar water can have stronger interaction with the surrounding than the cations. Considering the influence of the hydrophilic/hydrophobic properties of ions on the interactions between ions and surroundings, *MolLogP* was selected into the feature set.

MR (MolMR): *MolMR* (Molecular Refractivity) quantifies the overall polarizability or the ability of a molecule to respond to electric fields. It provides an estimation of the molecular size and shape. In *RDKit*, the calculation of *MolMR* is based on the method proposed by Wildman and Crippen[9]. It involves summing the atomic contributions of each atom in the molecule, where each atom's contribution is determined by its atomic polarizability value. The atomic polarizability values are pre-calculated and assigned based

on atom types. *MolMR* reflects the molecular properties related to van der Waals forces, dispersion forces, and polarizability, which may have an effect on the heat of transport of ions and thus on the magnitude of the Seebeck coefficient. Therefore, *MolMR* was selected as an input feature.

fh (fr_halogen): The *fr_halogen* is a molecular descriptor that represents the count of halogen atoms present in a molecule. In *RDKit*, the calculation of *fr_halogen* involves identifying the halogen atoms in the molecule based on their atomic symbols (F, Cl, Br, I) and counting the total number of occurrences. It was reported that by adding TPFPB, which contains halogen atom F, to the original i-TE system, the transport of cations will be hindered so that the Seebeck coefficient can be shifted from positive to negative (+20 to $-6 mVK^{-1}$)[11]. In view of that halogens can influence the electronic structure, polarity, and intermolecular interactions of a compound, *fr_halogen* was put into the feature set.

Note 2. Feature selection

Two primary methods were employed in the present study for feature selection, namely, wrapper methods and filter methods. Wrapper methods employ the performance of the machine learning model as a criterion to select the most promising features. Specifically, we implemented the recursive feature elimination wrapper method utilizing the pre-trained GBDT model as the wrapped model. This technique typically selects features that exhibit the highest feature importance. On the other hand, filter methods employ statistical measures to rank the features based on their relevance to the outcome variable. In this research, we employed a mutual information-based univariate selection method. Mutual information

calculates the dependence between two variables, and in this context, it was computed between each feature and the target variable, namely S_i, where the features with the highest mutual information were considered to be the most relevant.

Note 3. Algorithm screening

Performing algorithmic screening before detailed hyperparameter optimization is necessary for machine learning to ensure that the selected algorithm is appropriate for the specific problem at hand. Different algorithms are designed for different types of problems and data structures, and they have varying strengths and weaknesses. Therefore, we evaluated the performance of 18 models based on different algorithms. The training and test sets of the 18 models are identical, test set ratio being 0.2, and the hyperparameters are the default parameters in the *Scikit-learn* library. Table S1 presents the R^2 values of the 18 models ranked from highest to lowest on the test set. The results presented in Table S1 illustrate that the linear models (Linear Regression, Ridge, Lasso, Bayesian Ridge, ARD Regression, and SGD Regression) do not deliver promising results in terms of predictions. This observation may be attributed to the low probability of a linear relationship between the input features and S_i. Support vector machine (SVR, Nu SVR and Linear SVR), Gaussian Process Regression and K Neighbors Regressor models that can solve nonlinear problems perform relatively well. However, the ensemble models, namely XGBoost, RF, and GBDT, have exhibited the most superior predictive power. Therefore, these three models have been selected for further hyperparameter optimization. Additionally, the aforementioned models used only default hyperparameters for training and prediction in Scikit-learn, but a complex tuning process is

necessary for Artificial Neural Network (ANN) models which have been shown to successfully handle problems with small data sets[12,13]. Therefore, we have included ANN in the next step of the hyperparameter optimization process.

Note 4. Experimental section

Preparation of WPU/KI-x ionogels: WPU/KI-x ionogels, where 'x' represents the weight percentage of potassium iodide (KI) relative to the total weight of WPU and KI, were fabricated using ultrasonic separation. Initially, KI was dissolved in deionized water and stirred for 2 minutes at room temperature. Subsequently, the WPU emulsion was gradually introduced into the KI solution, stirring continuously for 5 minutes. The blend was then sonicated for 40 minutes to promote solid-liquid separation. It is essential to maintain the sonication temperature at or below ambient conditions. The WPU/KI ionogel was finally isolated by retrieving the solid component and draining off any excess water.

Materials: Waterborne polyurethane (WPU, product number-A909856), a polyether-based anionic water dispersion containing 40 wt% solids, was purchased from Macklin. Potassium iodide, cesium iodide, and sodium iodide were purchased from MERYER. All chemicals and materials were utilized as received without further purification. The electrode material, a hydrophilic carbon paper electrode (product number-BAC0437), was purchased from Beijing Zhongke Yannuo New Material Technology Co., Ltd.

Seebeck coefficient measurement: Open-circuit voltages were recorded at different temperature differences. The ionic thermovoltage was measured in terms of the Equation S2:

$$S_i = \frac{\Delta U \times L_2}{\Delta T \times L_1} \tag{2}$$

where ΔU represents the open circuit voltage, ΔT denotes temperature difference measured by the thermalcouples, and L_1 and L_2 are the distances between the two electrodes and two thermal couples, respectively. The Seebeck coefficients were determined by linear regression of the data across different temperature differences.

Note 5. Model interpretation

Permutation feature importance quantifies the impact of permuting feature values on the prediction error. A feature is considered important if shuffling its values leads to an increase in model error, indicating its significance in the prediction process. The Alibi package[14] is utilized to calculate permutation feature importance. For tree-based models such as Gradient Boosting Decision Trees (GBDT), feature importance can be evaluated by measuring the improvement in the performance metric at each feature split point, weighted by the number of observations assigned to the node. Tree-based feature importance is obtained using the *Scikit-learn* library. SHAP (Shapley additive explanations) values are calculated by assigning each feature value a weight that represents its contribution to the prediction. These values are computed using a method based on cooperative game theory, which measures the marginal contribution of each feature value in different coalitions of features. The SHAP package[15] in Python is used to calculate and average the SHAP values across all samples for a specific feature, providing insights into the individual feature's importance and its impact on the model's predictions. Plots illustrating the accumulated local effects are generated using the Alibi package.

Note 6. Calculation of diffusion coefficients and adsorption energies

As shown in Fig. S17, the amorphous cell module was used to build the simulation cells of the mixture including 20 polymer molecules and 20 ionic donors. Then, a geometry optimization was performed, and an annealing analysis was used to eliminate the energy barriers of the blend structure trajectories, additional reduced local hot spots, and thermal hysteresis[16]. Next, the annealing output configurations were taken as the initial configurations in the NVT simulation with a total simulation time of 5 ns. Following this, 10 ns simulation in the NVE ensemble was performed to acquire the mean square displacement (MSD) of the cations. For each configuration, the MSDs were calculated three times with different initial voltages and the average is used for the calculation of the diffusion coefficient. The diffusion coefficient of cations, D_{ca}, can be calculated using MSD as,

$$D_{ca} = \frac{\mathrm{MSD}}{2d\Delta t}, \tag{3}$$

where $d = 3$ indicates the dimension, and Δt is the time interval for the simulation of MSD.

The adsorption energy, E_{abs}, between cations and polymers were calculated by recording the energy variation when the cation and polymer molecule are freely adsorbed with each other and are separated. During the adsorption, the configurations of cation and polymer molecule are optimized with an energy threshold of 2.0×10^{-5} kcal/mol and a force threshold of 0.001 kcal/mol/Å. Then, when calculating the energy of separating configuration, the all atoms in cation and in polymer molecule keep distances > 20 Å. The structures of the eight cations adsorbed with PVA are shown in Fig. S18.

Note 7. Computational methods

Classical all-atom MD simulations were conducted using the Gromacs software (version 2022.7.4)[17]. The initial configuration for each system were established by Packmol[18]. The system comprised 100 polymer chains, 500 potassium ions, 500 iodide ions, and 5000 water molecules arranged randomly within a cubic box of 20 nm side length. Structural optimization was carried out using the Generalized Amber Force Field (GAFF)[19]. Water molecules were modeled using the three-point rigid OPC water model (OPC3)[20]. Temperature and pressure were maintained using the V-rescale thermostat[21] and the Parrinello-Rahman barostat[22], respectively. Long-range electrostatic interactions were computed using the particle-mesh Ewald (PME) method[23], with both electrostatic and Lennard-Jones interactions considered up to a cutoff distance of 1.3 nm. Simulations employed a time step of 2 fs, and visualizations were created with the VMD software[24]. The structure of the WPU was fully optimized at the B3LYP-D3BJ/6-311G** level of theory.

Supplementary Figures

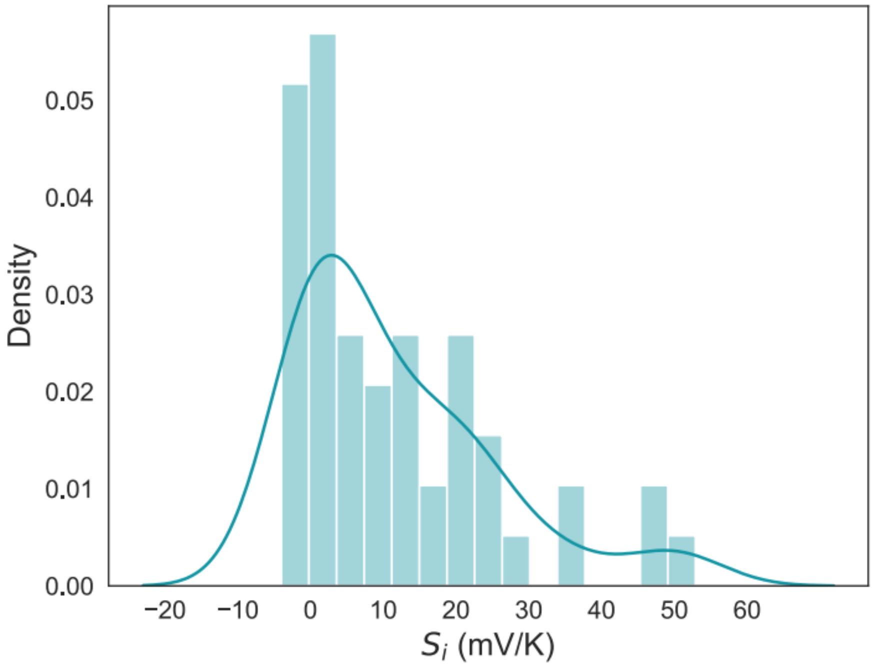

Fig. S1. S_i kernel distribution of totally 51 *i*-TE materials.

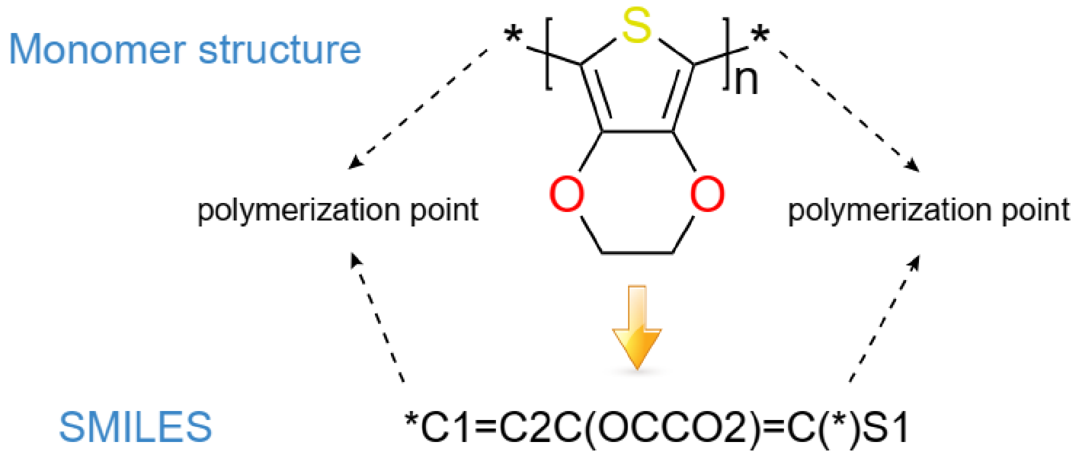

Fig. S2. An example of the representation of polymer matrix by SMILES encoding.

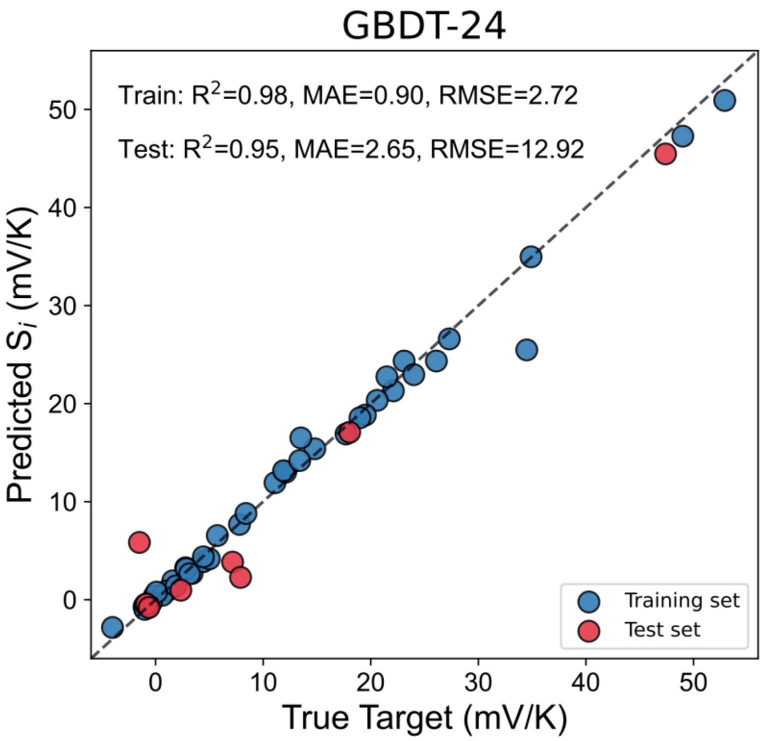

Fig. S3. The prediction results of the pre-trained model GBDT-24.

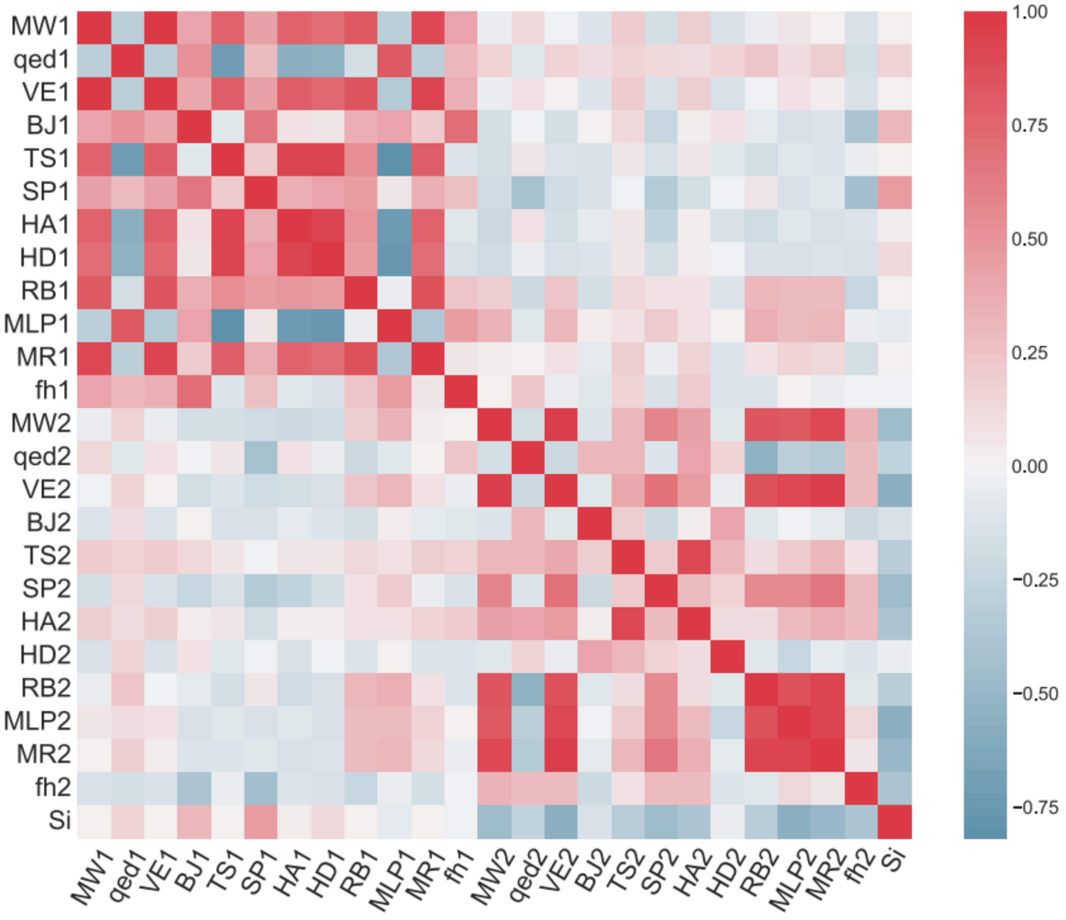

Fig. S4. Pearson correlation of 24 input features and S_i.

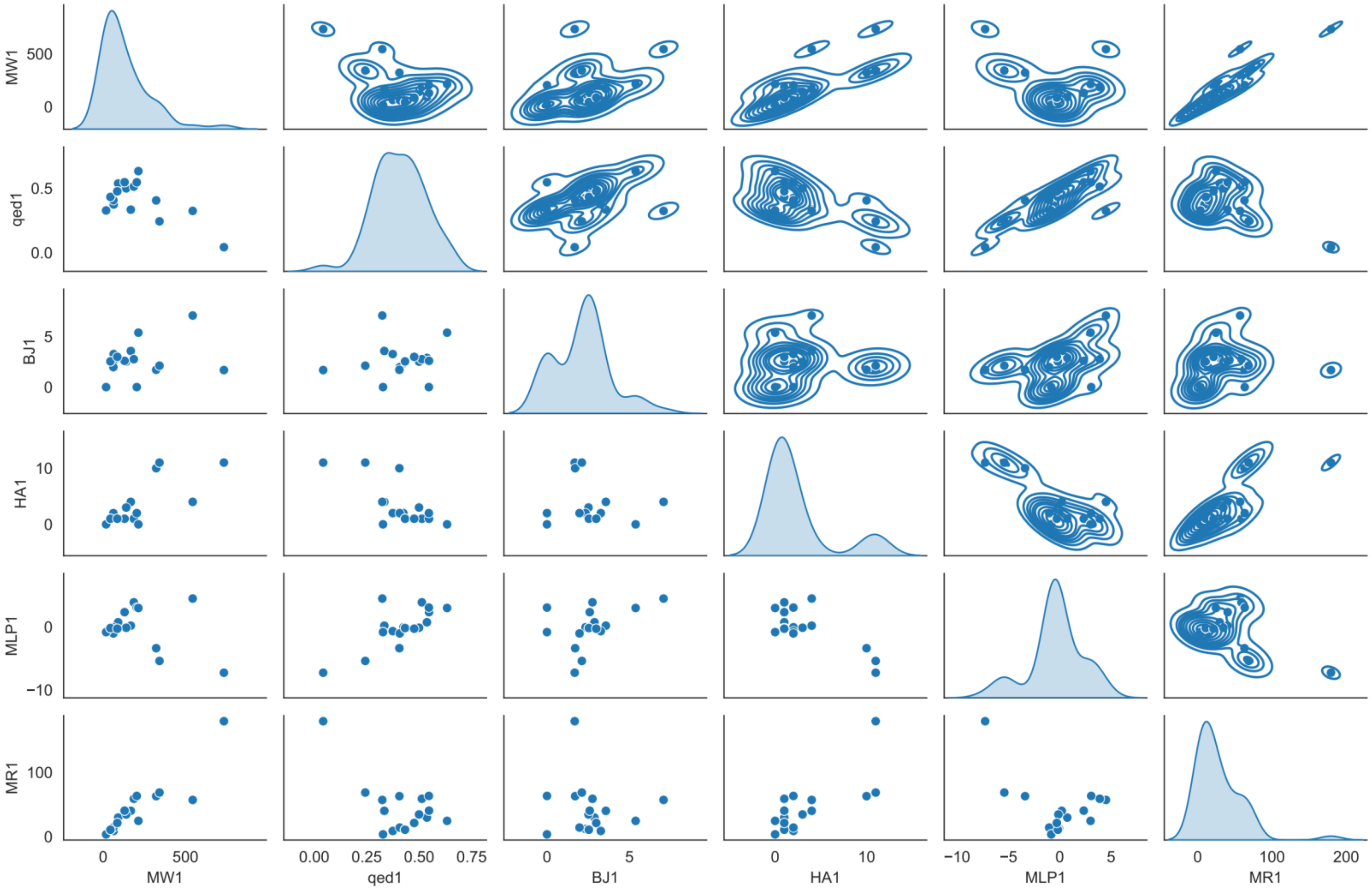

Fig. S5. Scatterplot that summarizes the joint distribution of the six selected properties of matrix materials.

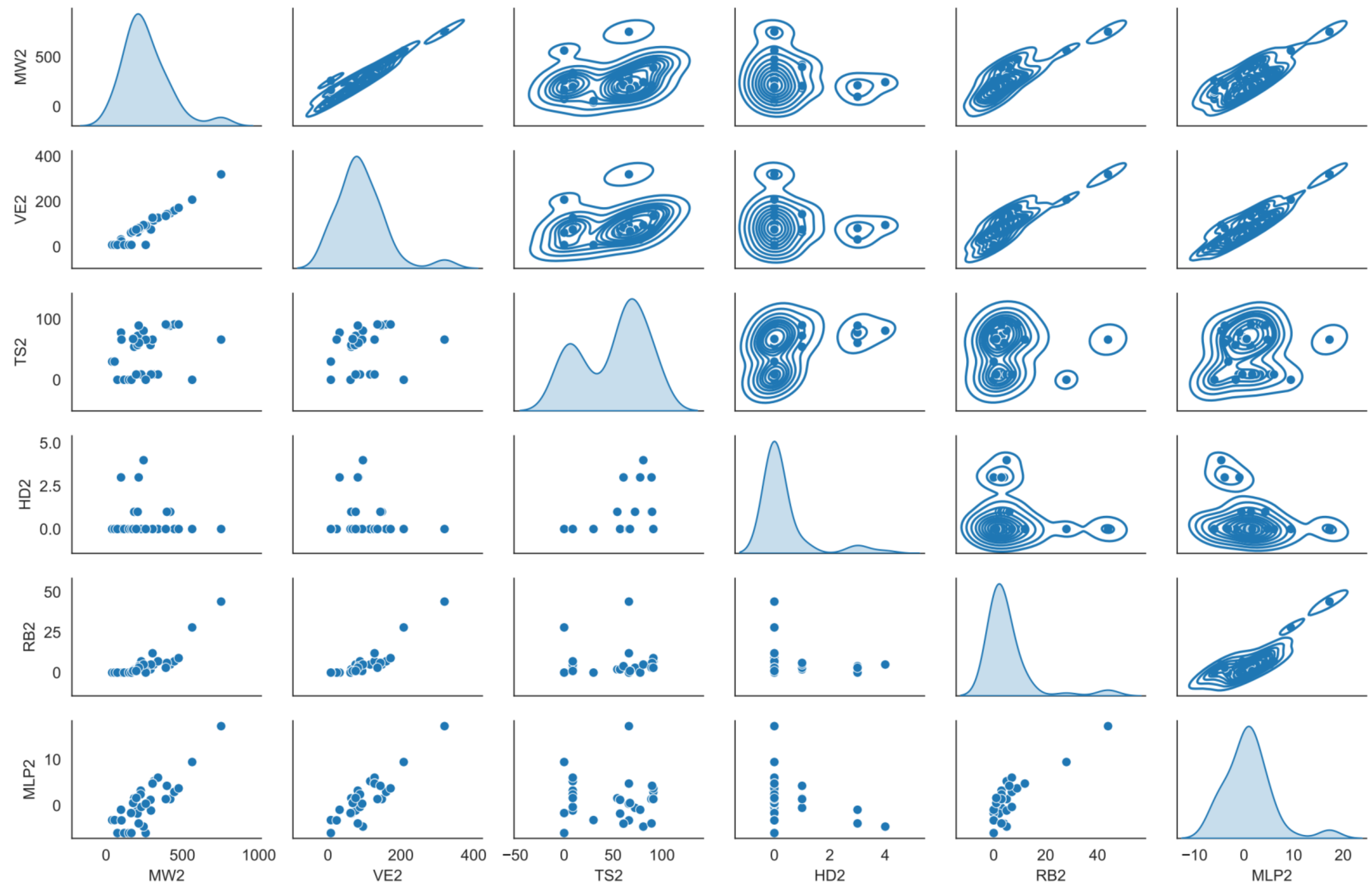

Fig. S6. Scatterplot that summarizes the joint distribution of the six selected properties of ion donors.

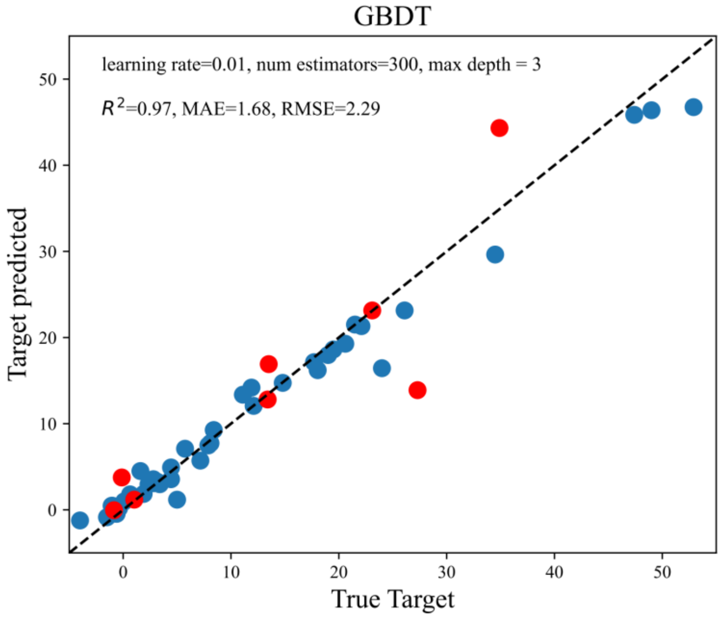

Fig. S7. The GBDT-24 model retrained with 12 selected features.

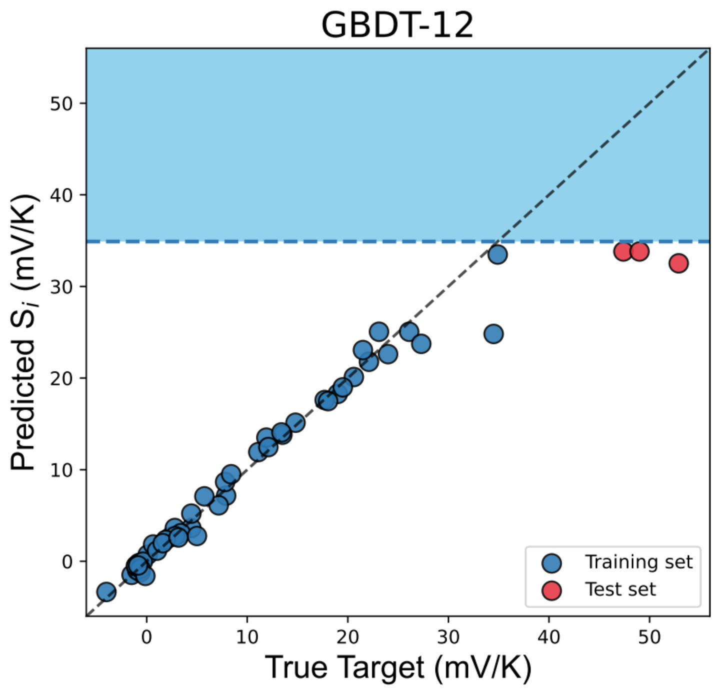

Fig. S8. The prediction results of the GBDT-12 model using a training dataset containing samples exhibiting Seebeck coefficients below 40 mV/K. The blue region is inaccessible to the test set dots, according to the algorithm principle of GBDT.

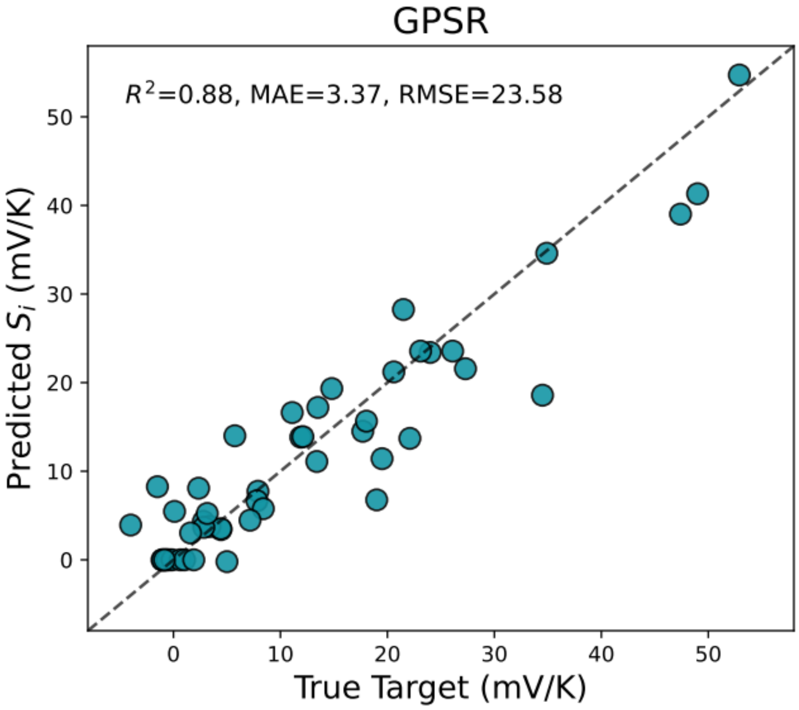

Fig. S9. GPSR aided forecast of Seebeck coefficients.

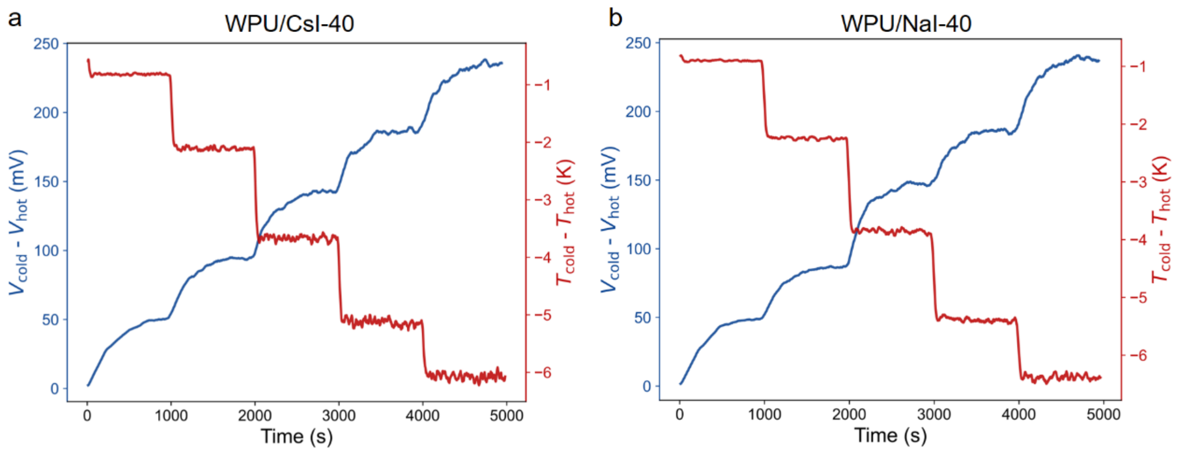

Fig. S10. The plots of the measured ΔV-ΔT curves of **a,** WPU/CsI-40 and **b,** WPU/NaI-40.

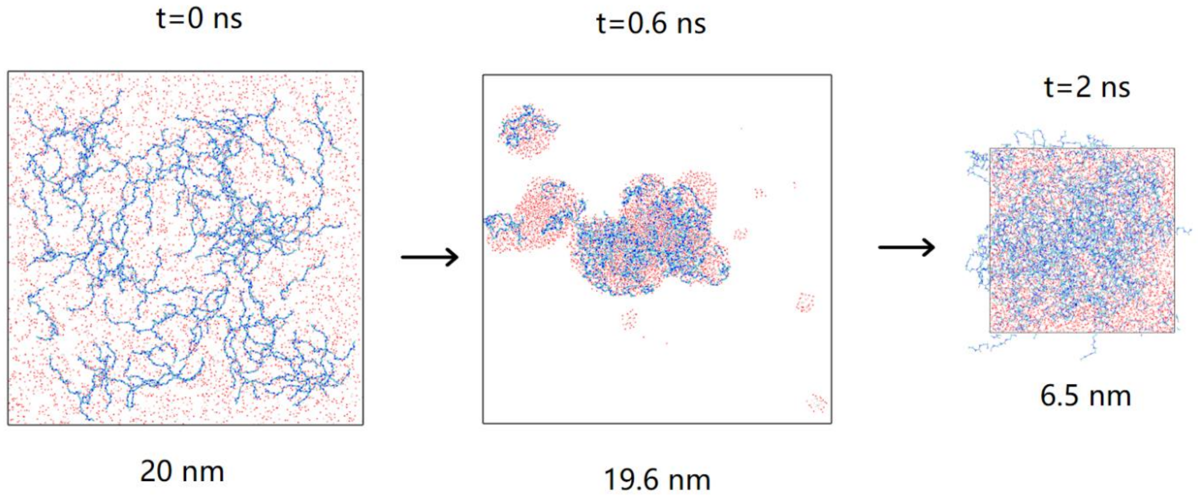

Fig. S11. Snapshots of different moments in MD simulations.

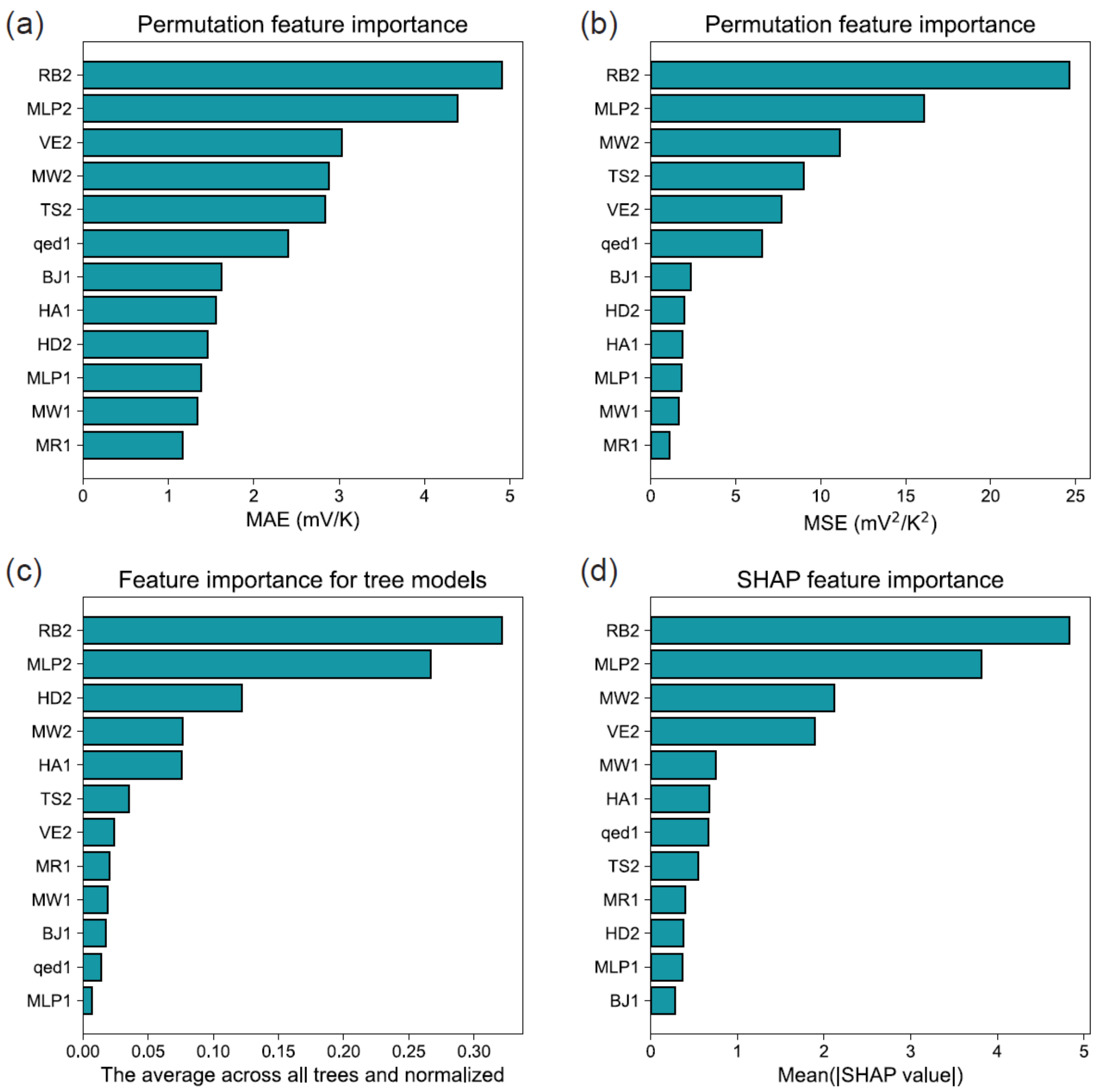

Fig. S12. Permutation feature importance using **a** MAE and **b** MSE as loss functions. **c**, Feature importance for tree models. **d**, SHAP feature importance.

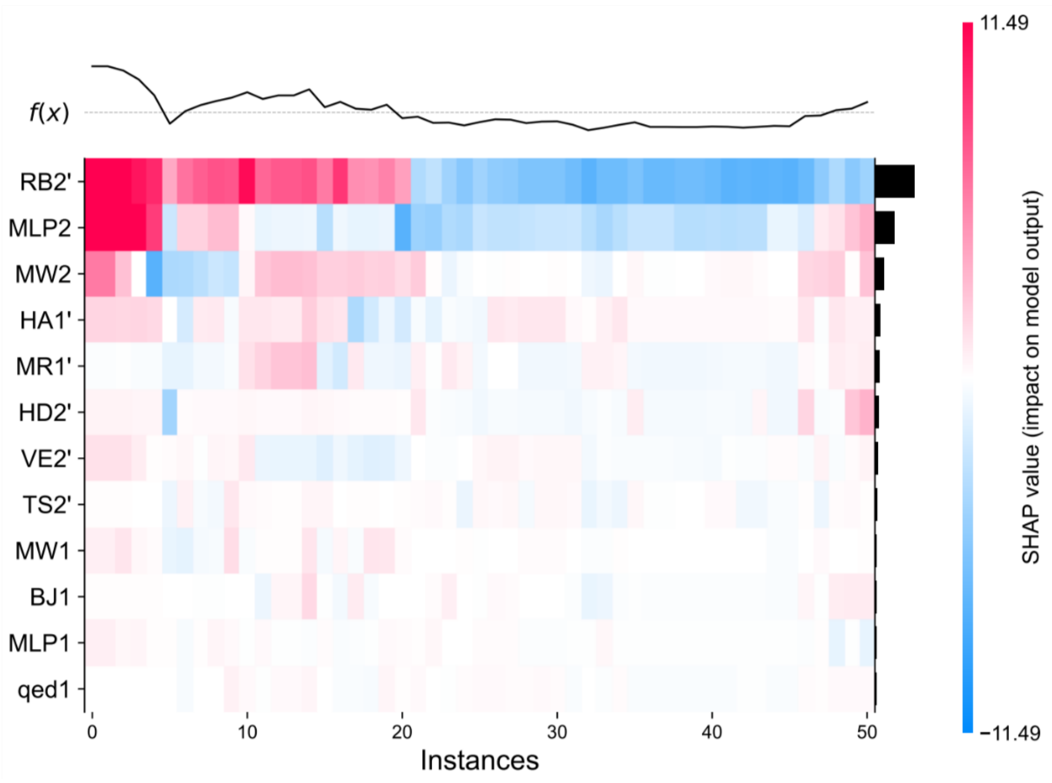

Fig. S13. The plot of SHAP heatmap, where the columns correspond to individual data points (instances) and each row represents a feature.

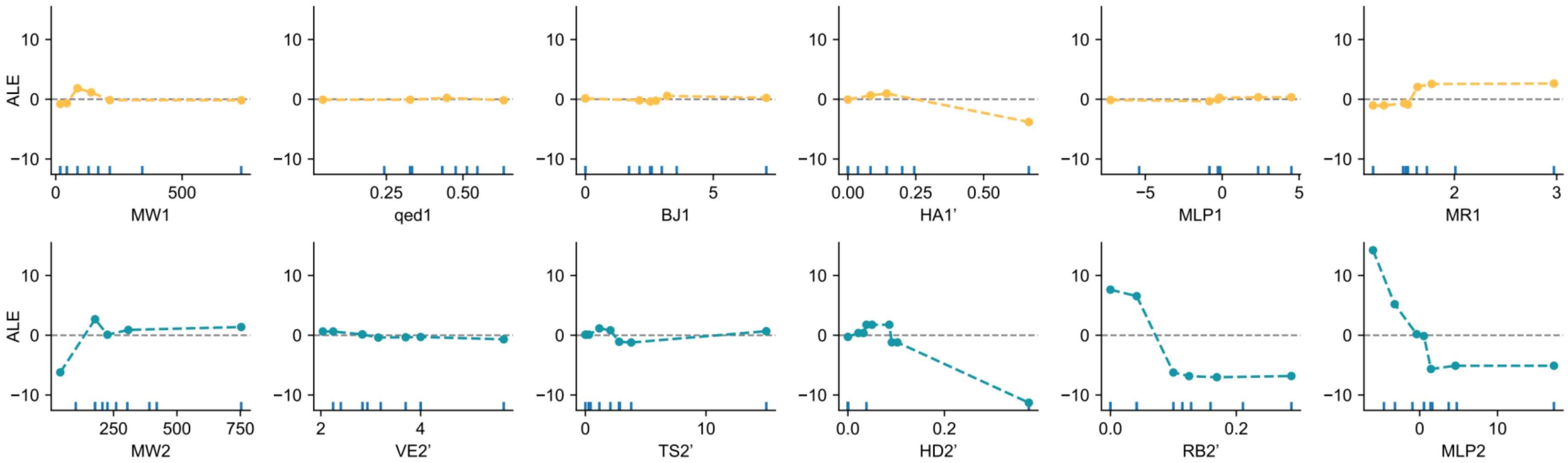

Fig. S14. Accumulated local effects (ALE) chart of the twelve features.

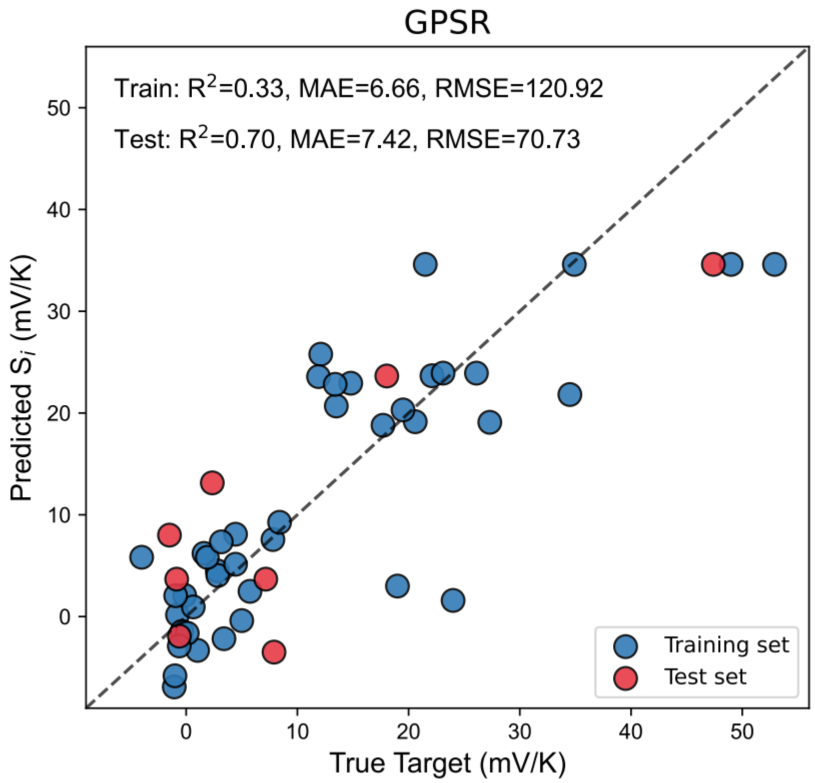

Fig. S15. Plots of the predicted versus true values of Seebeck coefficient by the GPSR model.

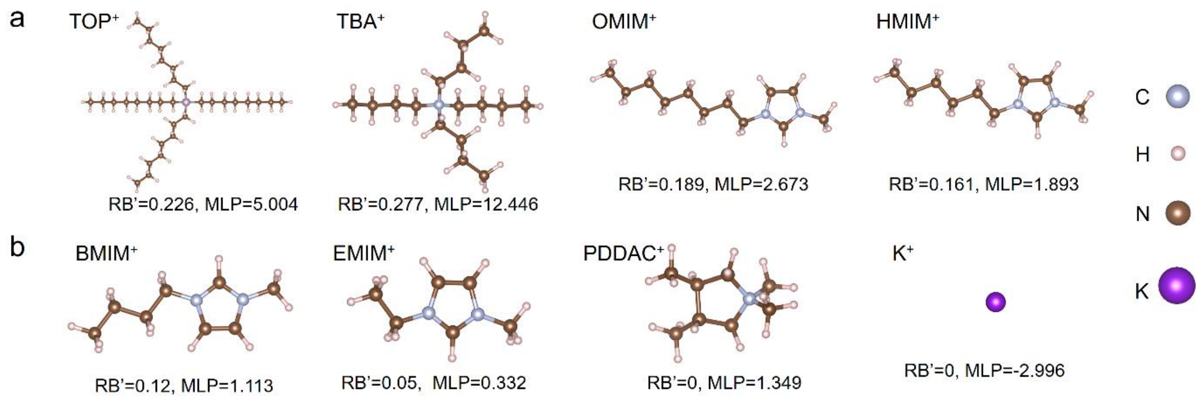

Fig. S16. Typical cations that resulting in higher and lower ionic Seebeck coefficient respectively, where the RB and MLP values are marked.

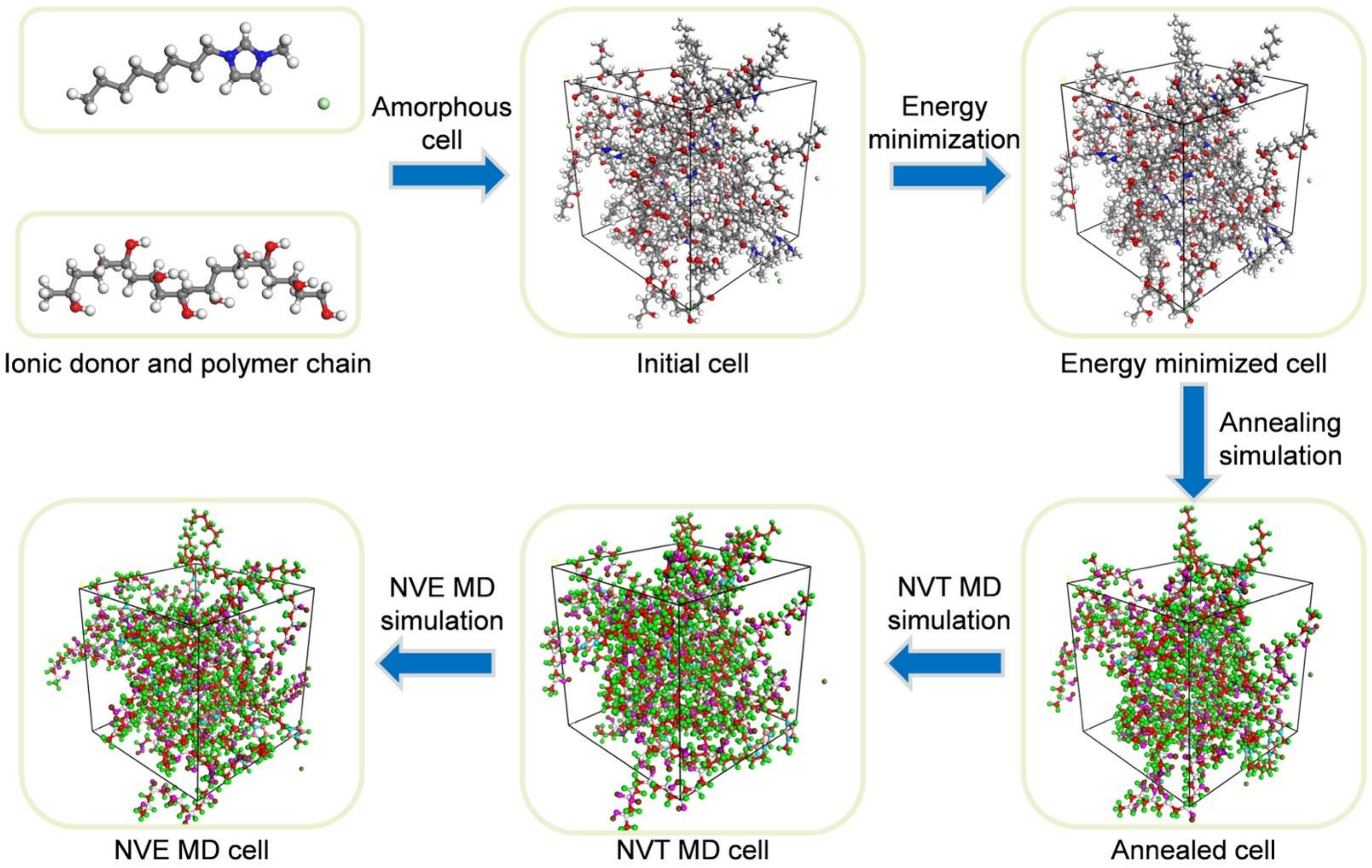

Fig. S17. Schematic of the steps of model construction and molecular dynamics simulations for the mixture of polymers and ionic donors. The simulation undergoes three steps including anneal simulation (T = 100 K ~ 300 K, ΔT = 10 K), NVT simulation (T = 300 K), and NVE simulation (T = 300 K).

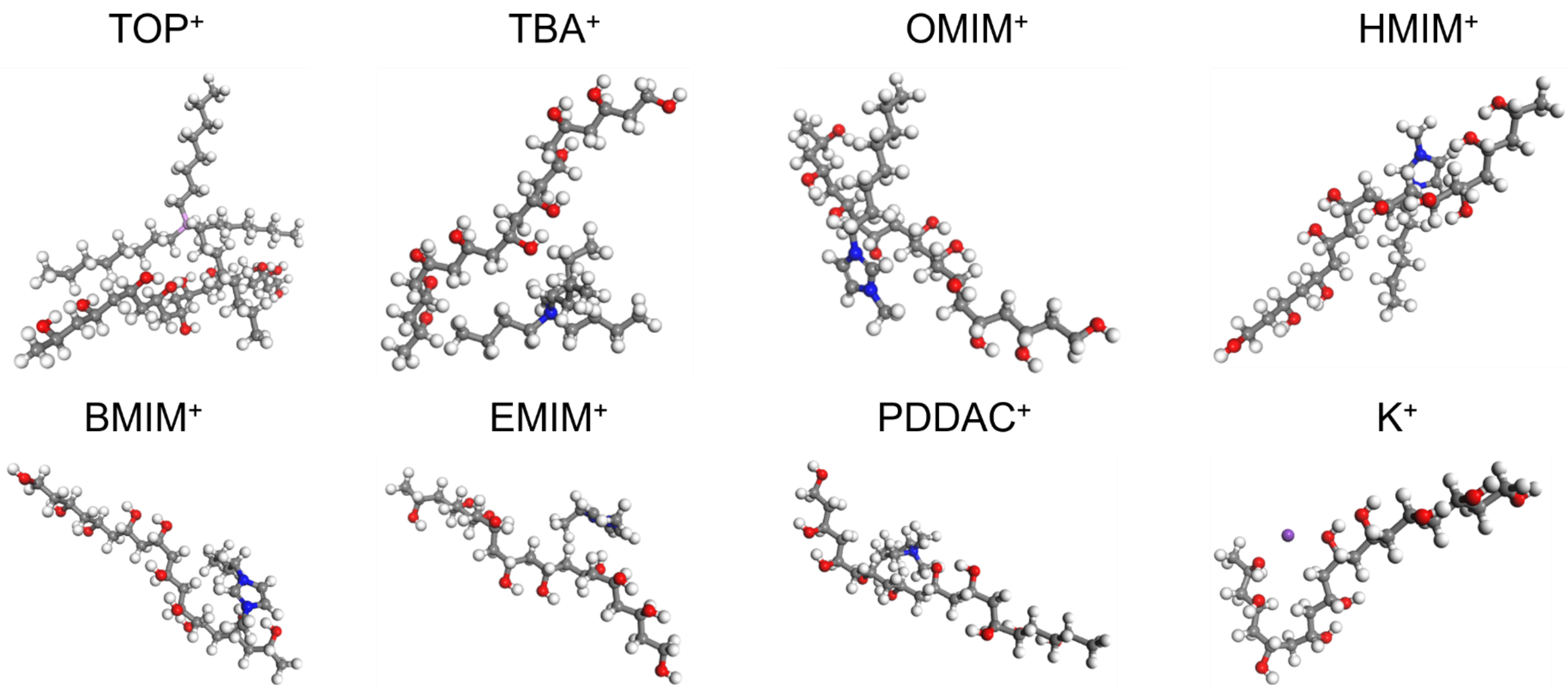

Fig. S18. Adsorption configurations of cations with PVA.

Supplementary Tables

Table S1. Statistical parameters in the training and test sets for 18 models.

Models	Training set				Test set			
	R^2	MAE	RMSE	MedAE	R^2	MAE	RMSE	MedAE
XGB Regressor	1.00	0.01	0.11	0.01	0.58	2.06	46.07	2.06
Random Forest Regressor	0.93	2.26	15.56	2.26	0.56	3.30	47.44	3.30
Gradient Boosting Regressor	1.00	0.20	0.16	0.20	0.50	2.56	54.56	2.56
Decision Tree Regressor	0.89	0.83	22.22	0.83	0.47	1.54	57.29	1.54
Extra Trees Regressor	0.91	3.04	18.87	3.04	0.39	3.10	66.09	3.10
Support Vector Regressor (SVR)	0.24	5.62	159.23	5.62	0.15	4.97	92.27	4.97
ANN	0.67	4.59	68.97	4.59	0.12	6.03	96.14	6.03
Nu SVR	0.19	8.48	170.64	8.48	0.10	6.84	97.57	6.84
K Neighbors Regressor	0.72	3.95	59.00	3.95	0.09	4.20	99.20	4.20
Gaussian Process Regressor	1.00	0.00	0.11	0.00	-0.15	8.82	124.58	8.82
Linear SVR	0.52	3.78	101.17	3.78	-1.04	6.58	221.58	6.58
Linear Regression	0.74	3.64	54.59	3.64	-1.25	11.40	244.54	11.40
Lasso	0.67	3.86	68.35	3.86	-1.86	12.07	311.42	12.07
Bayesian Ridge	0.71	3.56	61.24	3.56	-1.97	12.06	323.10	12.06
Ridge	0.73	3.88	55.97	3.88	-1.98	12.60	324.20	12.60
SGD Regressor	0.73	3.35	55.72	3.35	-2.16	12.84	343.38	12.84
ARD Regression	0.70	4.32	64.04	4.32	-2.52	13.07	382.58	13.07
PLS Regression	0.63	5.42	78.50	5.42	-2.93	16.00	427.01	16.00

Table S2. The setup of hyperparameters in sklearn for ANN-12

Hyperparameter	Value
hidden_layer_sizes	(6,)
alpha	0.054563264
activation	"tanh"
learning_rate	"constant"
learning_rate_init	0.169803684

Table S3. The setup of hyperparameters in sklearn for RF-12

Hyperparameter	Value
max_depth	4
max_features	"auto"
min_impurity_decrease	0

Table S4. The setup of hyperparameters in sklearn for GBDT-12

Hyperparameter	Value
criterion	"friedman_mse"
learning_rate	0.17
loss	"huber"
max_depth	5
max_features	"auto"
min_impurity_decrease	4
n_estimators	160
subsample	0.7

Table S5. The setup of hyperparameters in sklearn for XGBoost-12

**Hyperparameter**	**Value**
learning_rate	0.15
max_depth	6
n_estimators	180
subsample	0.8
booster	"gbtree"
colsample_bytree	1
colsample_bynode	0
gamma	0.8
min_child_weight	0

Table S6. Statistical parameters in the training and test sets for the 4 models under 10-fold cross-validation.

**Models**	**Training set**		**Test set**	
	**MAE (mV/K)**	**RMSE (mV/K)**	**MAE (mV/K)**	**RMSE (mV/K)**
ANN-12	0.932	1.576	7.339	9.390
RF-12	2.498	3.429	5.876	7.583
GBDT-12	0.900	1.440	4.885	6.225
XGBoost-12	0.793	1.051	5.447	6.555

Table S7. The ten matrix-ion donor combinations with the largest Seebeck coefficients predicted by GBDT-12 and XGBoost-12. The last column is the GPSR model-assisted judgment result.

Rank	Matrix	Ion donor	GPSR prediction (mV/K)
1	WPU	KI	31.294
2	WPU	CsI	39.111
3	WPU	NaI	29.951
4	PANI	CsI	36.197
5	SiO_2	KI	128.454
6	PANI	KI	28.981
7	octanol	CsI	23.792
8	SiO_2	CsI	190.992
9	SiO_2	NaI	117.715
10	dodecanol	CsI	17.565

Supplementary References

1 Bickerton, G. R., Paolini, G. V., Besnard, J., Muresan, S. & Hopkins, A. L. Quantifying the chemical beauty of drugs. *Nature chemistry* **4**, 90-98 (2012).

2 Balaban, A. T. Topological indices based on topological distances in molecular graphs. *Pure and Applied Chemistry* **55**, 199-206 (1983).

3 Ertl, P., Rohde, B. & Selzer, P. Fast calculation of molecular polar surface area as a sum of fragment-based contributions and its application to the prediction of drug transport properties. *Journal of Medicinal Chemistry* **43**, 3714-3717 (2000).

4 Li, T. *et al.* Cellulose ionic conductors with high differential thermal voltage for low-grade heat harvesting. *Nature Materials* **18**, 608-613 (2019).

5 Cheng, H., He, X., Fan, Z. & Ouyang, J. Flexible quasi-solid state ionogels with remarkable seebeck coefficient and high thermoelectric properties. *Advanced Energy Materials* **9**, 1901085 (2019).

6 Liu, S. *et al.* Giant and bidirectionally tunable thermopower in nonaqueous ionogels enabled by selective ion doping. *Science Advances* **8**, eabj3019 (2022).

7 Moriguchi, I., Hirono, S., Liu, Q., NAKAGOME, I. & MATSUSHITA, Y. Simple method of calculating octanol/water partition coefficient. *Chemical and Pharmaceutical Bulletin* **40**, 127-130 (1992).

8 Moriguchi, I., Hirono, S., Nakagome, I. & Hirano, H. Comparison of reliability of log P values for drugs calculated by several methods. *Chemical and Pharmaceutical Bulletin* **42**, 976-978 (1994).

9 Wildman, S. A. & Crippen, G. M. Prediction of physicochemical parameters by atomic

contributions. *Journal of Chemical Information and Computer Sciences* **39**, 868-873 (1999).

10 Cheng, H. & Ouyang, J. Soret effect of ionic liquid gels for thermoelectric conversion. *The Journal of Physical Chemistry Letters* **13**, 10830-10842 (2022).

11 Chi, C. *et al.* Selectively tuning ionic thermopower in all-solid-state flexible polymer composites for thermal sensing. *Nature Communications* **13**, 221 (2022).

12 Cha, G.-W., Moon, H. J. & Kim, Y.-C. A hybrid machine-learning model for predicting the waste generation rate of building demolition projects. *Journal of Cleaner Production* **375**, 134096 (2022).

13 Ma, X., Li, Z., Achenie, L. E. & Xin, H. Machine-learning-augmented chemisorption model for CO2 electroreduction catalyst screening. *The Journal of Physical Chemistry Letters* **6**, 3528-3533 (2015).

14 Klaise, J., Van Looveren, A., Vacanti, G. & Coca, A. Alibi explain: Algorithms for explaining machine learning models. *The Journal of Machine Learning Research* **22**, 8194-8200 (2021).

15 Lundberg, S. M. & Lee, S.-I. A unified approach to interpreting model predictions. *Advances in Neural Information Processing Systems* **30** (2017).

16 Salahshoori, I., Seyfaee, A., Babapoor, A., Neville, F. & Moreno-Atanasio, R. Evaluation of the effect of silica nanoparticles, temperature and pressure on the performance of PSF/PEG/SiO2 mixed matrix membranes: A molecular dynamics simulation (MD) and design of experiments (DOE) study. *Journal of Molecular Liquids* **333**, 115957 (2021).

17 Abraham, M. J. *et al.* GROMACS: High performance molecular simulations through multi-level parallelism from laptops to supercomputers. *SoftwareX* **1**, 19-25 (2015).

18 Mart ńez, L., Andrade, R., Birgin, E. G. & Mart ńez, J. M. PACKMOL: A package for building initial configurations for molecular dynamics simulations. *Journal of Computational Chemistry* **30**, 2157-2164 (2009).

19 Wang, J., Wolf, R. M., Caldwell, J. W., Kollman, P. A. & Case, D. A. Development and testing of a general amber force field. *Journal of Computational Chemistry* **25**, 1157-1174 (2004).

20 Izadi, S. & Onufriev, A. V. Accuracy limit of rigid 3-point water models. *The Journal of Chemical Physics* **145** (2016).

21 Bussi, G., Donadio, D. & Parrinello, M. Canonical sampling through velocity rescaling. *The Journal of Chemical Physics* **126** (2007).

22 Parrinello, M. & Rahman, A. Polymorphic transitions in single crystals: A new molecular dynamics method. *Journal of Applied physics* **52**, 7182-7190 (1981).

23 Essmann, U. *et al.* A smooth particle mesh Ewald method. *The Journal of Chemical Physics* **103**, 8577-8593 (1995).

24 Humphrey, W., Dalke, A. & Schulten, K. VMD: visual molecular dynamics. *Journal of Molecular Graphics* **14**, 33-38 (1996).
