# Supplementary material for: Unlocking new possibilities in ionic thermoelectric materials: a machine learning perspective
Source: Natl Sci Rev. 2024 Nov 23;12(1):nwae411. doi: 10.1093/nsr/nwae411 (PMC11702661; doi:10.1093/nsr/nwae411)
Supplement: nwae411_Supplemental_File [file nwae411_supplemental_file.zip › Teaser text.docx]

This work uses machine learning to predict the Seebeck coefficients of ionic thermoelectric materials, offering a faster path to discovering high-performance materials for efficient energy conversion.
